# Effects of wind energy generation and white-nose syndrome on the viability of the Indiana bat

**DOI:** 10.7717/peerj.2830

**Published:** 2016-12-22

**Authors:** Richard A. Erickson, Wayne E. Thogmartin, Jay E. Diffendorfer, Robin E. Russell, Jennifer A. Szymanski

**Affiliations:** 1 Upper Midwest Environmental Sciences Center, United States Geological Survey, La Crosse, WI, United States; 2 Geosciences and Environmental Change Science Center, United States Geological Survey, Denver, CO, United States; 3 National Wildlife Health Center, United States Geological Survey, Madison, WI, United States; 4 Division of Endangered Species, United States Fish and Wildlife Service, Onalaska, WI, United States

**Keywords:** Endangered species assessment, Full-annual-cycle, Migratory connectivity, Wind turbine mortality, White-nose syndrome, Population assessment, Indiana bat, *Myotis sodalis*

## Abstract

Wind energy generation holds the potential to adversely affect wildlife populations. Species-wide effects are difficult to study and few, if any, studies examine effects of wind energy generation on any species across its entire range. One species that may be affected by wind energy generation is the endangered Indiana bat (*Myotis sodalis*), which is found in the eastern and midwestern United States. In addition to mortality from wind energy generation, the species also faces range-wide threats from the emerging infectious fungal disease, white-nose syndrome (WNS). White-nose syndrome, caused by *Pseudogymnoascus destructans*, disturbs hibernating bats leading to high levels of mortality. We used a spatially explicit full-annual-cycle model to investigate how wind turbine mortality and WNS may singly and then together affect population dynamics of this species. In the simulation, wind turbine mortality impacted the metapopulation dynamics of the species by causing extirpation of some of the smaller winter colonies. In general, effects of wind turbines were localized and focused on specific spatial subpopulations. Conversely, WNS had a depressive effect on the species across its range. Wind turbine mortality interacted with WNS and together these stressors had a larger impact than would be expected from either alone, principally because these stressors together act to reduce species abundance across the spectrum of population sizes. Our findings illustrate the importance of not only prioritizing the protection of large winter colonies as is currently done, but also of protecting metapopulation dynamics and migratory connectivity.

## Introduction

**Figure 1:**
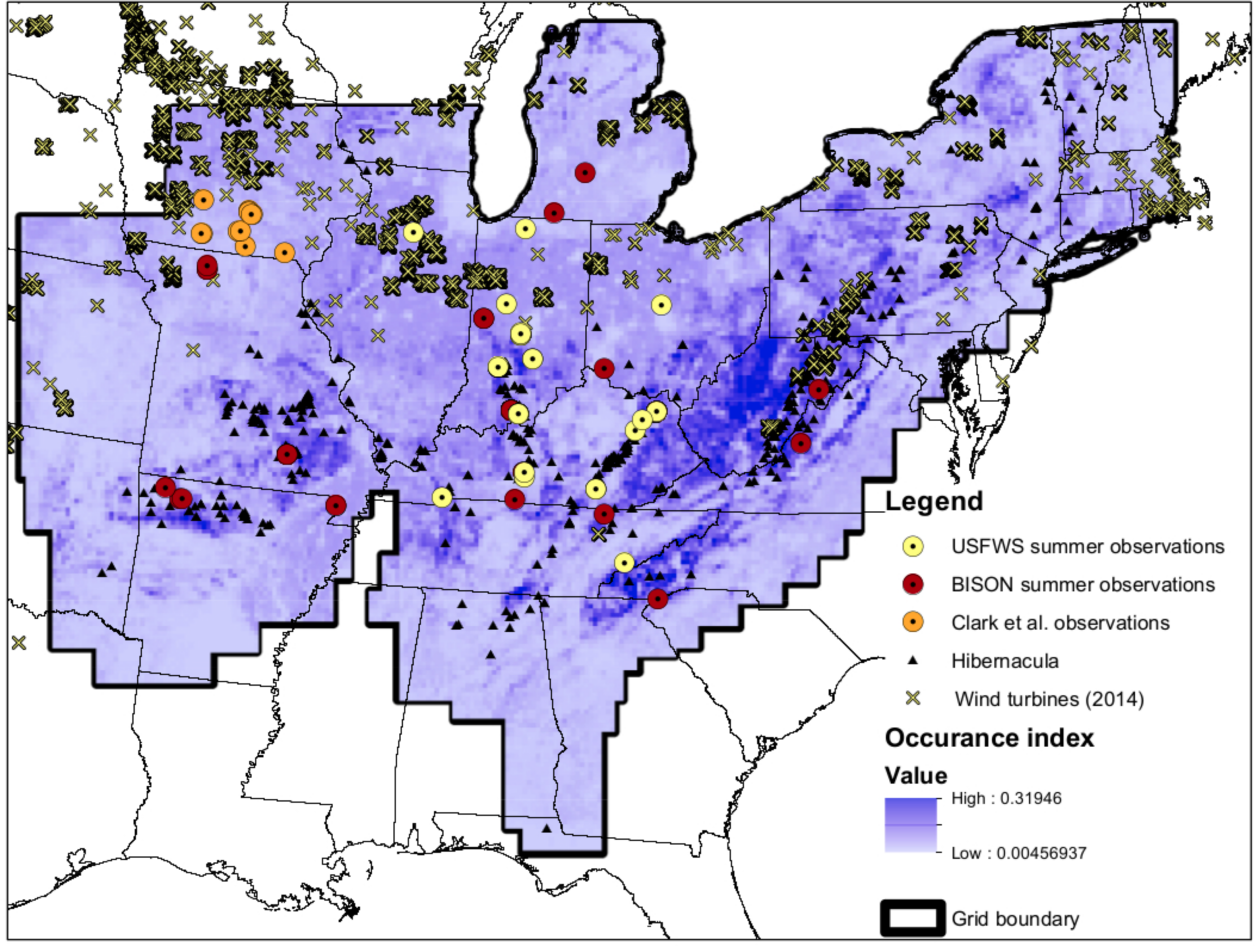
Map of input data. Map of input data, species occurrence map, and Indiana Bat species range. “USFWS summer observations” are from the US Fish and Wildlife Service. All US Fish and Wildlife Service data are from the endangered species program and exact locations are confidential. “BISON summer observations” are from the Biodiversity Information Serving Our Nation database (BISON; http://bison.usgs.ornl.gov). “Clark et al. observations” are capture data from Clark, Bowles & Clark (1987) “Hibernacula” data refers the winter hibernacula data from the US Fish and Wildlife Service. “Wind turbine data (2014)” comes from Diffendorfer et al. (2014). The white-to-blue color gradient depicts low to high suitability from the occurrence model. The grid boundary is the outline of the grid cells used for the occurrence model.

Wind energy generation holds potential as an alternative energy source to fossil fuels but also poses new threats to wildlife (Kuvlesky et al., 2007). In addition to the loss of habitat associated with wind turbine placement, collisions with wind turbines may cause mortality during migration (Kunz et al., 2007; National Research Council: Committee on the Status of Pollinators in North America, 2007; Arnett et al., 2008). One species possibly facing threats from wind energy is the Indiana bat (*Myotis sodalis*), an endangered species found in the midwestern and eastern United States (Fig. 1). The Indiana bat migrates seasonally between maternity colonies and hibernacula (caves and mines where the species overwinters), exposing the species to differential seasonal risk to wind energy (Pruitt & TeWinkel, 2007; Piorkowski et al., 2012).

Full-annual-cycle (FAC) models include mortality and reproduction for all seasons of a species life cycle and differ from traditional models that lump all seasons together (Hostetler, Sillett & Marra, 2015). Taylor & Norris (2010) developed an avian FAC model that has been applied to Mexican free-tailed bats (Wiederholt et al., 2013) and a theoretical model of *Myotis* spp. (Erickson et al., 2014). Traditionally, most migration models focus on summer and winter habitat use (e.g., breeding and non-breeding sites for migratory birds or maternity and hibernating sites for migratory cave bats) rather than migratory pathways (Taylor & Norris, 2010). Modeling migratory pathways, however, is critical to understanding the effects of mortality from wind energy generation on migrating wildlife. Here we apply the theoretical model developed by Erickson et al. (2014) to the entire range of the Indiana bat so that we may assess how current wind energy development may affect the species.

The Indiana bat was one of the first species listed under the Endangered Species Act of 1973. This act was passed with the goal of protecting the natural heritage of the United States of America and allows for plants and animals to be listed as either endangered or threatened (Office of the Federal Register, 1973). Although the original listing did not specify a reason, the consensus among bat experts was that human disturbance of hibernating bats caused population declines, prompting the listing (Pruitt & TeWinkel, 2007; Office of the Federal Register, 1967). Besides wind turbines (Langwig et al., 2012; Arnett & Baerwald, 2013), the species also faces threats from white-nose syndrome (Thogmartin et al., 2013) and hibernaculum vandalism (Crimmins et al., 2014), as well as broad threats from climate change, habitat loss, and land use change (Pruitt & TeWinkel, 2007; Loeb & Winters, 2012; Weber & Sparks, 2013). Populations of the species appeared to be recovering prior to the arrival of white-nose syndrome (WNS) (Thogmartin et al., 2012a), but declined as the disease spread (Turner, Reeder & Coleman, 2011). Recent research suggests these declines may not be as severe as initially feared, but concerns remain for species existence (Thogmartin et al., 2013; Powers et al., 2015).

White-nose syndrome affects cave bats such as the Indiana bat during hibernation and may cause up to 100% mortality, resulting in extirpations of local populations (Turner, Reeder & Coleman, 2011; Frick et al., 2015). *Pseudogymnoascus destructans*, the fungal causative agent of WNS, appears to opportunistically infect bats during hibernation (i.e., infects bats when their immune systems are less active during hibernation induced torpor) (Langwig et al., 2015). After infection, WNS initiates a physiological cascade of disturbances that often leads to the death of bats (Willis et al., 2011; Cryan et al., 2013; Warnecke et al., 2013; Verant et al., 2014). Prior to the arrival of WNS, no demographic population models (e.g., matrix population models in contrast to statistical population models) existed for any bat species (Hallam & Federico, 2009), and post-WNS arrival, modeling efforts have largely ignored spatial connections between populations (e.g., Thogmartin et al., 2012a; Frick et al., 2010). Conversely, the spatial model developed by Erickson et al. (2014) did not consider WNS and also did not model the observed spatial arrangement of populations.

Both wind energy development and WNS are spatially explicit threats to the Indiana bat. Wind turbines primarily affect Indiana bats along their migration routes (6 of 7 documented kills have been during the fall or spring; http://www.fws.gov/midwest/wind/wildlifeimpacts/inbafatalities.html#Table1), while WNS affects the survival of individuals overwintering in caves and mines. The effects of these two stressors have not been jointly studied for the Indiana bat, or for any bat species on a range-wide scale (American Wind Wildlife Institute, 2014). Herein, we examine the population-level effects of wind energy on the Indiana bat across its entire range. We also study the interaction between wind energy and WNS to understand whether the magnitude of mortality from wind may be sufficient to preclude recovery or increase risk of extirpation.

## Methods

We used a FAC model to explore potential impacts of wind energy development on the Indiana bat. Our model included data from multiple sources, including habitat and wind turbine data (Fig. 1). We described our model using the Overview, Design Concepts, and Details protocol (Grimm et al., 2006; Grimm et al., 2010) as part of our transparent and comprehensive model “evaludation” (TRACE) documentation (Schmolke et al., 2010; Augusiak, Van den Brink & Grimm, 2014; Grimm et al., 2014) (Supplemental Information 3). We also include our code as Supplemental Information 4 and our data have been published to a USGS webpage (Erickson, 2016). Within the remainder of this section, we provide an overview of our modeling approach and description of the data used within this approach.

The core of our population model is a series of difference equations (previously described in Erickson et al. (2014) and listed in our TRACE documentation). The model keeps track of groups of female Indiana bats using a pathway between a hibernaculum and a maternity colony. Erickson et al. (2014) formulated the model to include density at both maternity sites and hibernacula following Taylor & Norris (2010). We modified the model to only include density at the maternity colonies, which affected a baseline survival rate. We made this modification because hibernacula are unlikely to be limiting the Indian bat population sizes. Specifically, the total Indiana bat population appears to be at least one order of magnitude lower than pre-European settlement sizes and the number of hibernacula has remaining relatively stable or increasing through time as bats colonize old mines (Pruitt & TeWinkel, 2007).

We based our life history parameters upon previous models (Thogmartin et al., 2012b; Erickson, Thogmartin & Szymanski, 2014) and selected parameter values so that the annual population growth rate was 1.02 without the density effect. This value is concordant with pre-WNS growing Indiana bat populations (Thogmartin et al., 2013; Thogmartin et al., 2012a; Thogmartin et al., 2012b).

Our model landscape covered much of the eastern United States (Fig. 1). The landscape was divided into approximately 33,000 6500-ha grid cells because this resolution is considered to be equivalent to the home range area of an Indiana bat maternity colony by the USFWS (J Szymanski, pers. obs.). Furthermore, all hibernacula in close proximity (<10 km) are considered one unit for management by the USFWS (Pruitt & TeWinkel, 2007). The center of each grid cell containing hibernacula was connected to the center of all other grid cells to create migratory pathways. We assumed a maximum migration distance of 500 km, because most documented Indiana bat migration routes appear to be a shorter distance than this (Gardner & Cook, 2002; Winhold & Kurta, 2006). We excluded all “empty” hibernacula (i.e., those historically occupied, but now empty) from our model. We only used the highest 20% of summer maternity grid cells based upon the probability of Indiana bat occurrence, which is described in the next paragraph (Fig. 1). This left us with approximately 50,000 possible pathways between hibernacula and high quality maternity grid cells. Bats were placed on the model landscape at a random subset of hibernacula during model initialization. In any given run, approximately 5,000 of the 50,000 pathways were occupied.

Habitat occurrence of maternity sites for each grid cell was modeled with a logistic regression. We built upon previous work to identify covariates to consider (Loeb & Winters, 2012; Weber & Sparks, 2013; Farmer, Cade & Stauffer, 2002; Miller et al., 2002; Yates & Muzika, 2006). We initially compared several models that included different combinations of mean monthly temperature, different mean monthly precipitations, land cover, mean elevation, and maximum slope. We used the Watanabe Akaike information criterion (WAIC) for our model selection (Watanabe, 2010) because this method is fully Bayesian and considers parameter distributions unlike other model selection approaches (Gelman et al., 2013). We used Stan (version 2.4, http://www.mc-stan.org), as implemented through RStan, to fit our models and calculate the WAIC values (Hoffman & Gelman, 2014). Our final model included crop cover, deciduous forest cover, and May precipitation. The complete parameter values for this model are described in our TRACE documentation (Supplemental Information 3).

We used the WNS-spread Map from 12 March 2015 (https://www.whitenosesyndrome.org/resources/map) to model spread of the disease through time. We modeled WNS (the disease), rather than *Pseudogymnoascus destructans* (the fungus) because this is what the North American WNS response group tracks. We assumed that any hibernaculum without WNS would have WNS by 2016 because WNS has spread across the entire range of the Indiana bat. We adapted the white-nose syndrome model used in Thogmartin et al. (2012b) to be a continuous time function rather than a piecewise discrete function. We used a logistic function to describe bat survival from WNS through time (Bolker, 2008). The model depended upon the baseline winter survival rate and the arrival year of white-nose syndrome, and also included a slope term and intercept term. The intercept term is offset by the arrival year term to account for WNS arriving during different years and we included three different intercept terms to account different WNS survival scenarios. Parameter were based upon our parameterization as described in the TRACE Documentation (Supplemental Information 3).

**Figure 2:**
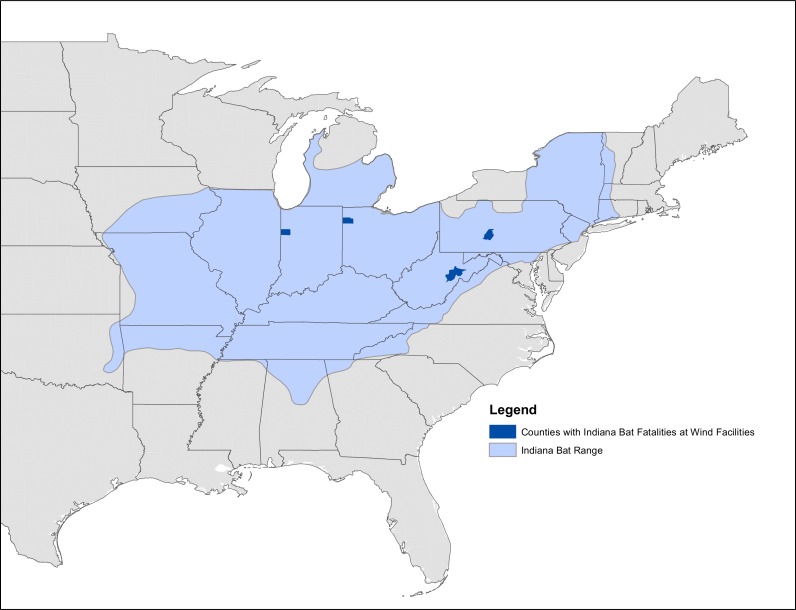
Map of known Indiana bat fatalities. Counties with known Indiana bat fatalities at wild facilities. The fatalities mapped are those known to the US Fish and Wildlife Service as of April 2015. The figure is from “Indiana Bat Fatalities at Wind Energy Facilities” by Lori Pruitt and Jennifer Okajima, US Fish and Wildlife Service, Indiana Field Office (http://www.fws.gov/midwest/wind/wildlifeimpacts/inbafatalities.html). The figure was created by US Government employees during their official duties and is therefore in the public domain.

We modeled the effect of wind turbines on the Indiana bat by decreasing survival based upon the number of turbines found along a migratory pathway or in a maternity site or hibernaculum grid cell. The number of turbines present decreased the baseline survival. Due to uncertainty about the number of bats killed by turbines (Arnett & Baerwald, 2013), we used three mortality scenarios: A low mortality scenario with 1 of 1,000 bats flying by a turbine killed, a medium mortality scenario with one out of 100 bats flying by a turbine killed, and a high mortality scenario with one out of 10 bats flying by a turbine killed. These three scenarios were chosen to bound the spectrum of possible responses and accommodate uncertainty in Indiana bat mortality at turbines, as well as include a “safety factor” consideration (Suter II, 2006). This uncertainty in mortality exists because, to date, only 7 Indiana bats have been reported killed at wind energy facilities to the USFWS (Fig. 2; http://www.fws.gov/midwest/wind/wildlifeimpacts/inbafatalities.html). The medium and high scenarios are unlikely to be an average (or “expected”) mortality rate for all migratory pathways, but represent worst case collisions risks for bats flying through turbines. The actual collision risk for any specific wind turbine and an Indiana bat likely depends upon many local environmental conditions that change temporally (e.g., wind direction during the few minutes that an Indiana bat is flying through a turbine farm). Using a stochastic collision risk would possibly improve model realism, but only if we had a meaningful distribution from which to draw.

Turbine location data were from Diffendorfer et al. (2014). Winter and summer mortality from wind turbines only considered the mortality from the cell containing the colony because of the species small home range during non-migratory seasons (Pruitt & TeWinkel, 2007). No hibernacula cells had turbines present within them. Each migratory pathway was buffered on each side by 1-km, 2-km, 10-km, and 20-km. This buffer distance accounted for uncertainty in the Indiana bat migration route. We focused on the 2-km buffer pathway (4-km wide) because USFWS experts consider this to be the most reasonable scenario (J Szymanski, pers. obs., 2013).

The model was programmed in **R** (R Core Team, 2014) using the data.table package (Dowle et al., 2014). We parallelized our code for the stochastic runs using the doSNOW package (Revolution Analytics & Weston, 2014). Our code is included as Supplemental Information 4.

## Results

**Figure 3:**
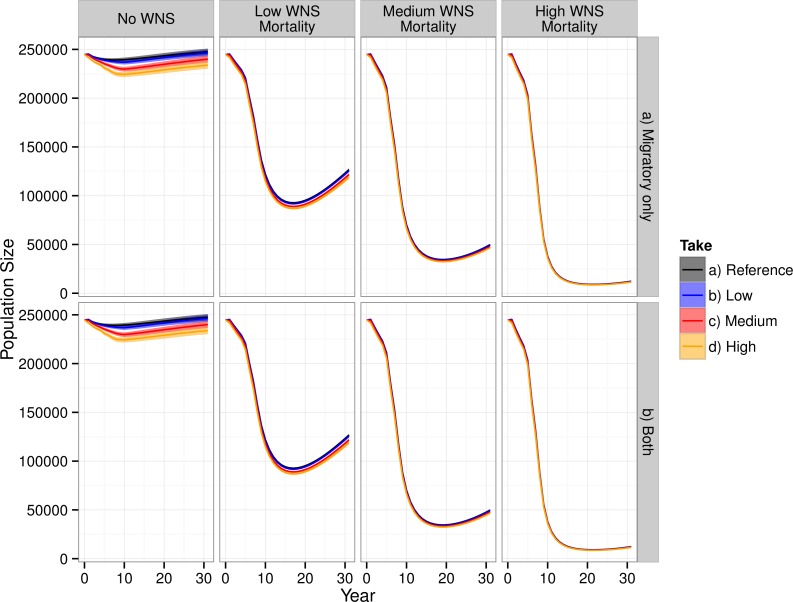
Total population of female Indiana bats predicted by the model. The figure is faceted on the *x*-axis by WNS mortality scenarios. The figure is faceted on the *y*-axis by wind turbine exposure scenarios. “*Migratory only*” refers to bats only being killed along the migration pathways whereas “*Both*” allows the bats to be killed at both the summer and winter habitats as well as along the migratory pathway. We only show the results from including turbines found within a 2-km buffer of the migratory pathway.

WNS had the largest impact on the modeled population dynamics of the Indiana bat (Fig. 3). The highest WNS mortality scenario caused a ≈95% decline, which caused extreme imperilment for the species. The medium WNS mortality scenario decreased the population size by ≈80% whereas the WNS mortality scenarios reduced the total population size by ≈50%. Uncertainty existed about where Indiana bats live and migrate on the landscape, which affected their mortality from wind turbines within the model and led to model uncertainty (i.e., a probabilistic output seen in the No WNS scenarios). However, the inclusion of WNS overwhelmed this spatial uncertainty and almost completely reduced the model’s uncertainty (i.e., the range of the resulting probability distribution declined to near zero).

Including WNS as part of the simulations appeared, at first glance, to overshadow the effects of wind turbine mortality. Wind turbine mortality affected the system in a nuanced and subtle manner. In the scenarios without WNS, the lowest wind turbine mortality rate caused a decline of less than 1% in total population size, the medium mortality rate caused a 3% decline, and the high mortality rate caused a 6% decline. These differences were reduced within the low WNS mortality scenarios and disappeared within the medium and high WNS mortality scenarios. This difference between WNS scenarios occurred because WNS killed bats that would have otherwise been killed by wind turbine strikes. We also found that turbine-caused mortality at colonies was negligible compared to mortality along the migratory pathways. Presumably, this is because there was little overlap between modeled summer maternity colonies and wind turbines and no overlap between known hibernaculum cells and wind turbines.

Despite killing fewer individuals than WNS, wind turbines affected the metapopulation dynamics of the Indiana bat more than WNS for all scenarios other than the high-WNS mortality scenario (Fig. 3). Without WNS, the low- and medium-wind mortality scenarios decreased the number of migration pathways by 6% whereas the high WNS mortality scenario caused almost all of the pathways to go extinct (<99%).

**Figure 4:**
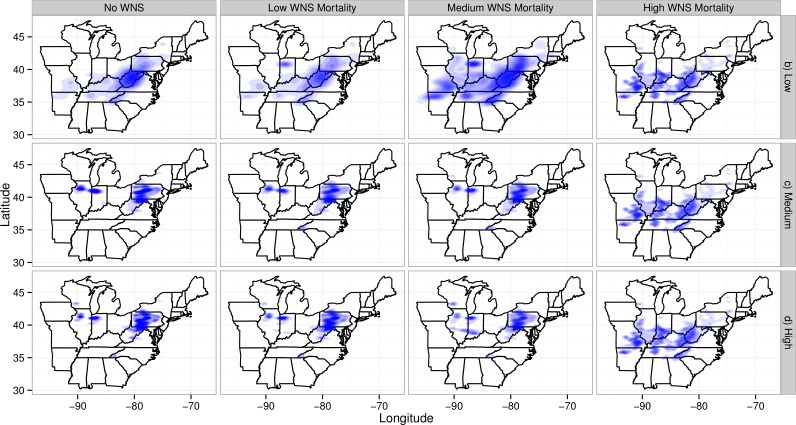
Map of maternity colonies lost under different exposure scenarios. The figure is faceted on the *x*-axis by different WNS mortality scenarios. The figure is faceted on the *y*-axis by different wind turbine mortality rates. We only show the results from including turbines found within a 2-km buffer of the migratory pathway. We also did not plot the scenarios that only included take occurring along migratory pathways. The shading is the relative density of colonies lost. The density is subplot specific and only qualitative comparisons should be made across subplots. Furthermore, the area and shading of the density varies across plots because of the the shading algorithm used by *ggplot2*. This plotting program shrinks the density as the number of points increases and the variability among points decreases.

The loss of migratory pathways corresponded to the loss of maternity colonies (Fig. 4) and winter colonies (Fig. S1). Wind turbines caused the loss of maternity colonies primarily in two clusters: one in northern Illinois and Indiana (the western cluster) and the second in the Appalachians of West Virginia and Pennsylvania (the eastern cluster). The western cluster corresponded to an area with moderate abundances of Indiana bat maternity colonies and high abundances of wind turbines. The eastern cluster corresponded to an area with high abundances of Indiana bat colonies and moderate abundances of wind turbines. As WNS mortality rates increased, the loss of maternity colonies shifted south and west. This corresponded to a shift from areas with wind turbines to an area where the majority of the winter colonies for the species have been found. It is also worth noting that some of the low wind turbine mortality scenarios have fewer and more dispersed deaths than other scenarios. The spatial density plots reflected the uncertainty, spatial variability, and number of mortalities in simulations (Wickham , 2009). Plots from scenarios with fewer mortalities and greater spatial variability would have larger shaded regions than plots from scenarios with more mortalities and less spatial variability because the first scenarios have more uncertainty in the confidence regions.

The extirpation of winter colonies followed a different pattern than the loss of maternity colonies (Fig. S1). Wind turbine mortalities led to the loss of three clusters of winter colonies. One cluster was in the Appalachian regions of Pennsylvania and West Virginia and was similar to the eastern cluster of maternity colonies lost, and was located near a high density of wind turbines. A second cluster occurred mostly in western Kentucky, whereas a third cluster occurred mostly in southern Missouri. These two clusters were not located near any wind energy generation facilities. As modeled WNS mortality rates increased, the locations of winter colonies lost tended to become more evenly distributed across the range of winter colonies.

Although we only compared 4 different wind turbine mortality levels, interpolation to different levels of mortality may be possible (Fig. S2). Our lowest level of wind turbine mortality had very little effect on the final total population size. A decline in population size occurred as mortality from wind increased between the low and medium wind turbine mortality scenarios. A smaller decline occurred between the medium and high wind turbine mortality scenarios. This smaller decline suggests a leveling off of mortality (e.g., a point of diminishing return), such that further increases in the mortality rate from wind turbines, as they are currently configured across the United States, would have little additional effect because wind turbines removed all groups affected by energy generation. Thus, the bats are depopulated from the turbine areas when mortality is high.

## Discussion

The current juxtaposition of wind energy facilities within the range of the Indiana bat may lead to a meaningful impact on the population dynamics of the species, depending upon the magnitude of risk from collision faced by bats in migration. Although wind energy may have some effect on the simulated total population size (Fig. 3), the effects of wind turbines on the metapopulation dynamics and, specifically, on migrational connectivity of the Indiana bat are likely more important owing to the reduction in number of migratory pathways within our model (Fig. S1). At the simulated rates of mortality from turbines, wind energy facilities hold the potential to extirpate smaller over-wintering populations (Barclay & Harder, 2003; Jones, Purvis & Gittleman, 2003). Survival of these smaller sub-populations is likely critical for the species to survive WNS because smaller winter colonies appear less at risk from WNS (Thogmartin et al., 2012b; Wilder et al., 2011). This finding also highlights important differences in compensatory and additive mortality. At the population-level, wind turbine development and white-nose syndrome appear to be compensatory sources of mortality (i.e., if wind turbines did not kill Indiana bats, white-nose syndrome would kill them anyways). However, when examining the two stressors together at the meta-population level, the two stressors quite likely are additive. Wind turbine mortality would be more likely to extirpate small hibernacula whereas white-nose syndrome would be more likely to extirpate large hibernacula. Our finding also raises concerns about wind turbines and WNS producing a synergistic effect on the population dynamics of the species, where each stressor has a much greater impact when considered jointly than would be expected from that stressor acting alone.

Current USFWS management of the Indiana bat focuses on protecting large winter colonies because most of the individual bats use a few caves (Pruitt & TeWinkel, 2007; Thogmartin & McKann, 2014). Additionally, current models used by the USFWS for issuing incidental take permits ignore the spatial structure of the population (Thogmartin et al., 2012b; Erickson, Thogmartin & Szymanski, 2014). The USFWS may benefit from explicitly considering metapopulation dynamics as WNS kills a growing portion of the population and wind energy production increases. Specifically, placing additional emphasis on protecting small winter colonies may be prudent (Thogmartin & McKann, 2014). Additionally, a more complete model for WNS might help guide conservation efforts because different risk factors appear to affect survival (Boyles & Willis, 2009; Flory et al., 2012; Wilder et al., 2011). Empirically quantifying and understanding these effects will be critical to understanding the dynamics of the species and the disease affecting it (Thogmartin et al., 2013).

Similar to WNS, a paucity of data exists for modeling how wind turbines affect Indiana bat survival. This deficit of data created some of the greatest uncertainty in our model because our wind turbine mortality scenarios varied by orders of magnitude. As of 2015, the USFWS has only received reports of seven Indiana bats being killed at wind turbine facilities (Fig. 2; http://www.fws.gov/midwest/wind/wildlifeimpacts/inbafatalities.html#Table1). Estimating the number of Indiana bats killed by wind turbines is difficult due to a lack of standardized protocols for sampling wind turbines for all species (Huso, 2011; Huso, 2013), hampering meta-analysis across study sites (Loss, Will & Marra, 2013; Beston, Diffendorfer & Loss, 2015). Additionally, the Indiana bat is difficult to find because it is a small species that decomposes quickly after death and is difficult to correctly identify (Arnett et al., 2011). Further, the no standardized reporting framework exists for wind energy mortality within the United States. A better understanding of the conditions under which turbines kill Indiana bats would not only allow a better understanding of the species population dynamics, but also allow for possible protective measures to be taken (Arnett et al., 2011).

The lack of data on wind turbine collision risk limits population-level assessments for all species, not just the Indiana bat (Loss, Will & Marra, 2013; Beston, Diffendorfer & Loss, 2015). To date, few studies (e.g., Carrete et al., 2009; Schaub, 2012) have examined range-wide effects of wind turbines on a specific species; ours is the first to look at multiple stressors at the population-level. Our findings illustrate how mortality from wind turbines interacts with other stressors. Modeled wind turbines strongly affected (and often extirpated) small sub-populations whereas modeled WNS caused a fairly uniform decline across the entire range. This result also demonstrates the need for greater understanding of compensatory mortality when examining incidental take (McGowan et al., 2011). Although our findings only examine one anthropogenic stressor affecting the stability of one species, similar trends are emerging world wide where anthropogenic stressors are affecting the stability, biodiversity, and productivity of Earth’s ecosystems (Dirzo et al., 2014; Harley, 2011; Hautier et al., 2015).

To capture the salient life history aspects of the Indiana bat, our FAC required significant effort at parameterization. This effort may be possible for other endangered species such as the whooping crane (*Grus americana*) (Nations, Howlin & Young, 2013), but is not easily scalable to the hundreds of species killed at wind turbines. For species where it is not possible to construct high-effort, high-input models, probably the first and most important question to ask would be, “what is the overlap between the species range and wind turbines?” As an example of such an assessment, Santos et al. (2013) applied spatial distribution modeling to examine four species of bats and what factors affected the probability of mortality occurring at a given wind energy production facility. Similarly, work by Roscioni et al. (2013) modeled the regional effects of wind farms on bats, and Roscioni et al. (2014) modeled the effects of wind farms on bat migration and population connectivity. As part of the spatial overlap question, it is also important to not only consider the “where,” but also the “when.” Obviously, a species with no overlap is not directly at risk, but might be if wind energy generation adversely affects an important competitor or prey species.

The other modeling efforts we described are similar in that they broadly seek to understand the impacts of wind energy development on wildlife. These efforts differed, however, in either their scale or modeling approaches. For example Nations, Howlin & Young (2013) constructed an individual-based model for an extremely rare species that would have more of a localized risk of wind turbine mortality. Santos et al. (2013) used species distribution models to examine spatial distribution and risk using distribution modeling rather than population modeling. Efforts by Roscioni et al. (2013); Roscioni et al. (2014) were similar to our in that they examined spatial migration and networks. Specifically, Roscioni et al. (2014) examined the spatial connectivity of a bat species in Italy and the possible effects of wind turbine development on the species. However, our approach differs from Roscioni et al. (2014) because we focused on the population dynamics of the species.

Another important consideration is the spatial structure of the population: “Are there distinct subpopulations or is the species well connected across its range?” The Indiana bat forms distinct subpopulations because of its life history, but other species such as long-distance migratory tree bats or some avian species may not. This spatial connectivity is also important if one decides to consider the possibility of re-colonization of extirpated populations. The third important consideration that emerges from our results would be, “What are the other stressors affecting the population and how do they interact with wind energy production?”

Due to the large number of parameters in and uncertainty within our model, additional research data and model improvement could be incorporated to refine our approach. More summer field observations of the Indiana bat would be especially beneficial. A North American Bat Monitoring Program is being developed, but does not currently have extensive data on the Indiana bat (Loeb et al., 2015). This data would allow more certainty in modeling of summer habitat, which would also allow for better understanding of migratory routes. Additionally, Pauli et al. (2015) recently modeled how different landscape management scenarios affect the Indiana bat and models such as theirs could be linked to population models if computational limits and data limitations could be overcome. Using an agent-based model approach, treating migratory groups as the agent, or an individual based-modeling approach, would also allow our model to capture more salient behaviors (Grimm & Railsback, 2005). Another important consideration is colonization and re-colonization metapopulation dynamics. Indiana bats have been observed colonizing abandoned mines (Pruitt & TeWinkel, 2007), which we did not explicitly consider. Lastly, our model did not consider demographic stochasticity or demographic heterogeneity (Melbourne & Hastings, 2008). We did not include these components because of computational limits. Modeling these would have required using natural numbers, but we used continuous numbers because it simplified the model and decreased computation time. Additionally, demographic stochasticity would require the use of either computationally intense methods such as binomial distributions or programming-intense methods such as branching process models (Caswell, 2001; Erickson et al., 2015).

## Conclusion

Under some of the modeled levels of influence, wind energy production may deleteriously affect the population dynamics of the Indiana bat. We found wind energy production’s effects were principally at the metapopulation-level and primarily affected smaller winter colonies. Combined with WNS, which principally affects larger colonies, management may need to consider metapopulation dynamics and focus on protecting smaller Indiana bat winter colonies to reduce risk of species extinction. Our findings also illustrate the broader importance of considering FACs and migratory networks rather than simply focusing on local habitat or homogeneously distributed range-wide populations.

##  Supplemental Information

10.7717/peerj.2830/supp-1 Supplemental Information 1 Meta data, model output data, and geospatial data Data was broken down into 2 files because of size limitations. The data will be published to a USGS webpage (most likely Science Base) concurrently with publication of the article. It is being provided for the reviewers and editors here.

10.7717/peerj.2830/supp-2 Supplemental Information 2 Model input data Data was broken down into 2 files because of size limitations. The data will be published to a USGS webpage (most likely Science Base) concurrently with publication of the article. It is being provided for the reviewers and editors here.

10.7717/peerj.2830/supp-3 Supplemental Information 3 TRACE documentation for the model

10.7717/peerj.2830/supp-4 Supplemental Information 4 Supporting Code

10.7717/peerj.2830/supp-5 Supplemental Information 5 Figures S1 & S2

## Additional Information and Declarations

### Data Availability

The following information was supplied regarding data availability: USGS: http://dx.doi.org/10.5066/F75M63TN.
