# Supplementary material for: Effects of wind energy generation and white-nose syndrome on the viability of the Indiana bat
Source: PeerJ. 2016 Dec 22;4:e2830. doi: 10.7717/peerj.2830 (PMC5183089; doi:10.7717/peerj.2830)
Supplement: Supplemental Information 1 — Data was broken down into 2 files because of size limitations. The data will be published to a USGS webpage (most likely Science Base) concurrently with publication of the article. It is being provided for the reviewers and editors here. [file peerj-04-2830-s001.zip › 2016-23 (Erickson, Indiana Bat Open Data )/FSP_form_data_2015_RAE_ibat.pdf]

UMESC
July 2015

FSP Review – Data Release

SCIENCE CENTER Tracking No.	INFORMATION PRODUCT Tracking No.

AUTHOR(S)

R.A. Erickson, W. Thogmartin, J. Diffendorfer, R. Russell, J. Szymanksi

TITLE

Data supplement for: Assessing the impacts of wind energy and white-nose syndromee on the Indian bat

SCIENCE CENTER CONTACT

JC Nelson
Upper Midwest Env. Sci. Ctr.
2630 Fanta Reed Road
La Crosse, WI 54603
608-781-6370
Fax: 608-783-6066
jcnelson@usgs.gov

BASIS+ PROJECT

No.

Task SubTask

LTRM TRACKING NO.

TYPE OF PRODUCT *(check all that apply)*

Geospatial	*Science*	*Other*
[x] Shapefile	[ ] Spreadsheet	[ ] Datasheets
[ ] Geodatabase	[ ] Database	[ ] Field/Project Notes
[ ] Raster	[ ] Text	[ ] Metadata *(only)*
[ ] Imagery	[ ] Images	[ ] Graphics or Maps
[ ] KML(Z)	[x] Other	[ ] Other
[ ] Other		

Type of Other→ CSV files

DISTRIBUTION METHOD

- [ ] UMESC Web
- [ ] ScienceBase
- [ ] Product to Partner
- [x] Other Web Site
- [ ] Other

Check all that apply

FSP REVIEW STATUS *(determined by Branch Chief)*

- [ ] Non-Interpretive
- [ ] Previously FSP Reviewed
- [ ] New Interpretive

If Previously FSP Reviewed, Location in the Science Files

No.

Task SubTask

BASIS No.
project, task, sub-task

Folder Color: [ ] *Yellow* [ ] *Purple* [ ] *Orange* [ ] *Blue* [ ] *Red*

Product Number

The searchable index of Science File contents is available at N:\UMESC Science Files

DISTRIBUTOR *-or-* NAME OF RECIPIENT

Related Manuscript IPDS Tracking No.

REMARKS *For more information see http://www.usgs.gov/datamanagement/share/datarelease.php*

NAME	DATE IN	DATE OUT	TASK	CHECK WHEN COMPLETE
1 Richard Erickson<br>Author		RICHARD ERICKSON<br>Digitally signed by RICHARD ERICKSON DN: c=US, o=U.S. Government, ou=Department of the Interior, ou=Geological Survey, cn=RICHARD ERICKSON, 0.9.2342.19200300.100.1.1=14001002924718 Date: 2015.07.30 13:29:39 -05'00'	[x] Email your supervisor requesting a FSP review<br>[x] Give network location of data	
2 Jack Waide<br>Branch Chief			[ ] Review product, determine readiness for FSP<br>[ ] Verify Interpretive vs Non-Interpretive http://www.usgs.gov/fsp/interpretive_definitions_and_examples.asp<br>[ ] Email Carol L.; approve the product for review	
3 Metadata Reviewer			[ ] Create a report that details any issues found<br>[ ] Return report to the Author	
4 Data Reviewer			[ ] Create a report that details any issues found<br>[ ] Return report to the Author	
5 Author			[ ] Update product [ ] Send to Supervisor/Copy Carol L.<br>[ ] Document reviewer reconciliations	
6 Branch Chief			[ ] Product check [ ] Send to Carol L. (**Only reviews and reconciliation loaded into IPDS**)<br>[ ] Reconciliation check	
7 Center Director			[ ] Reconciliation and Policy Check	
8 Bureau Approval (Only Interpretive)			[ ] Check comments and record in IPDS	
9 UMESC Data Manager			[ ] Obtain Web URL and Digital Object Identifier<br>[ ] Update metadata and put on-line	
