# Supplementary material for: Effects of wind energy generation and white-nose syndrome on the viability of the Indiana bat
Source: PeerJ. 2016 Dec 22;4:e2830. doi: 10.7717/peerj.2830 (PMC5183089; doi:10.7717/peerj.2830)
Supplement: Supplemental Information 3 [file peerj-04-2830-s003.pdf]

TRACE document

This is a TRACE documentation ("TRAnsparent and Comprehensive model Evaludation") which provides supporting evidence that our model presented in:

Richadr A. Erickson, Wayne E. Thogmartin, Jay E. Diffendorfer, Robin E. Russell, Jennifer A. Szymanksi. The synergistic effects of wind energy generation and white-nose syndrome threaten the extinction of the endangered Indiana bat. PeerJ.

was thoughtfully designed, correctly implemented, thoroughly tested, well understood, and appropriately used for its intended purpose.

The rationale of this document follows:

Schmolke A, Thorbek P, DeAngelis DL, Grimm V. 2010. Ecological modelling supporting environmental decision making: a strategy for the future. *Trends in Ecology and Evolution* 25:479-486.

and uses the updated standard terminology and document structure in:

Grimm V, Augusiak J, Focks A, Frank B, Gabsi F, Johnston, Liu C, Martin BT, Meli M, Radchuk V, Thorbek P, Railsback SF. 2014. Towards better modelling and decision support: documenting model development, testing, and analysis using TRACE. *Ecological Modelling* 280:129-139

and

Augusiak J, Van den Brink PJ, Grimm V. 2014. Merging validation and evaluation of ecological models to 'evaludation': a review of terminology and a practical approach. *Ecological Modelling.* 280:117-128

Contents

1 Problem Formulation **3**

2 Model description **4**

2.1 Purpose 5
2.2 Entities, state variables, and scales 5
2.3 Process overview and scheduling 6
2.4 Design concepts 6
2.4.1 Basic principles 6
2.4.2 Emergence 8
2.4.3 Adaptation 8
2.4.4 Objectives 8
2.4.5 Learning 8
2.4.6 Prediction 9
2.4.7 Sensing 9
2.4.8 Interaction 9
2.4.9 Stochasticity 9
2.4.10 Collectives 9
2.4.11 Observation 9
2.5 Initialization 9
2.6 Input data 10
2.6.1 Habitat data 10
2.6.2 Population model inputs 11
2.7 Submodels 11
2.7.1 Habitat occurrence model 11
2.7.2 Population model 13

3 Data evaluation **15**

4 Conceptual model evaluation **17**

5 Implementation verification **18**

6 Model output verification **19**

7 Model analysis **19**

8 Model output corroboration **21**

Bibliography **22**

1 Problem Formulation

This TRACE element provides supporting information on: The decision-making context in which the model will be used; the types of model clients or stakeholders addressed; a precise specification of the question(s) that should be answered with the model, including a specification of necessary model outputs; and a statement of the domain of applicability of the model, including the extent of acceptable extrapolations.
Summary:

The Indiana Bat, an endangered species found in the eastern United States, may be adversely affected by wind energy development. We constructed a population model to understand these effects and help resource managers guide recovery efforts. We also considered the effects of white-nose syndrome due to its large impact upon the species. Our model results cover the entire range of the Indiana Bat. Directly extrapolating the model to other species would not be possible, however, the model could be reparameterized for the other *Myotis* spp.

Our model was designed to understand the population-level effects of wind energy development on the Indiana Bat by researchers and managers (e.g., U.S. Fish and Wildlife Service Biologists who are leading recovery efforts for the species), however, any recovery efforts for the species must also consider the effects of white-nose syndrome (WNS) on the population. Both of these are spatially explicit stressors. A secondary target audience might include consultants who are involved with the wind energy industry and are concerned with population-level effects of wind turbines on bats.

We specifically used the model to ask to following questions:

1. What are the population-level effects of current wind energy development on the Indiana Bat?
2. How does wind energy development interact with white-nose syndrome when affecting Indiana Bat population dynamics?
3. What is the spatial distribution of wind energy's effects on the Indiana Bat?

In order to answer these questions, the model produced the number of hibernacula, number of maternity colonies, number of migratory routes, and total population size. These outputs were generated from different simulation conditions that modeled effects of white-nose syndrome and wind energy development. The location of hibernacula and maternity colonies lost during the simulation were also recorded to address our spatial question.

Our model was developed for the Indiana Bat and covers the entire known range of the species. No direct extrapolations would be possible with the model. The model could reasonably be applied to other *Myotis* spp. by changing the parameters and model inputs (i.e., colony locations) due to their similar life history (i.e., only producing one pup, overwintering in caves, migrating to summer maternity colonies). Similarly, other stressors could be included as part of the model by including their effect on the Indiana bat. Broadly, our model could be adapted to any species living more than one year if changed to be appropriate for the species (e.g., number of life stages).

2 Model description

This TRACE element provides supporting information on: The model. Provide a detailed written model description. For individual/agent-based and other simulation models, the ODD protocol is recommended as standard format. For complex submodels it should include concise explanations of the underlying rationale. Model users should learn what the model is, how it works, and what guided its design.

Summary:

Our Indiana Bat population-level model is described in detail. We present the model using the ODD format, which includes the habitat modeling approach we used.

2.1 Purpose . . . 5

2.2 Entities, state variables, and scales . . . 5

2.3 Process overview and scheduling . . . 6

2.4 Design concepts . . . 6

2.4.1 Basic principles . . . 6

2.4.2 Emergence . . . 8

2.4.3 Adaptation . . . 8

2.4.4 Objectives . . . 8

2.4.5 Learning . . . 8

2.4.6 Prediction . . . 9

2.4.7 Sensing . . . 9

2.4.8 Interaction . . . 9

2.4.9 Stochasticity . . . 9

2.4.10 Collectives . . . 9

2.4.11 Observation . . . 9

2.5 Initialization . . . 9

2.6 Input data . . . 10

2.6.1 Habitat data . . . 10

2.6.2 Population model inputs . . . 11

2.7 Submodels . . . 11

2.7.1 Habitat occurrence model . . . 11

2.7.2 Population model . . . 13

Overview

We applied a model developed by Taylor and Norris (2010) and Erickson et al. (2014a) to understand how wind energy development would affect the Indiana Bat. The model is a network model consisting of summer nodes (maternity sites) and winter nodes (hibernacula) connected by paths (migration routes). The model is also based upon the stage-structured Indiana Bat model developed by Thogmartin et al. (2012). The model differs from Taylor and Norris (2010) and Erickson et al. (2014a) by two important attributes. First, this model does not include density dependence within the hibernacula because Indiana Bats historically occurred at much greater densities than present levels (pre-European colonization abundances were at least one and likely more orders of magnitude greater than current population sizes). Second, this model does not include migration survival as a function of distance because our model is focused on the Indiana bat, which forages as it migrates as opposed to an avian species that does not forage as it migrates except at stopover sites. The model also differs from Taylor and Norris (2010) because it does not include arrival order within the model. Taylor and Norris (2010) included arrival order because it describes avian populations where the first migrants returning get the best nest sites, but cave bats live in colonies without fixed individual territories. Our model also differs from Erickson et al. (2014a) because we apply the model to an actual (versus theoretical) landscape.

Briefly, our modeling efforts consists of the following steps: 1) Parameterizing an occurrence model using ArcPy and other Python modules to manipulate data and RStan to parameterize the model, 2) Generating of a landscape by connecting hibernacula with summer sites, 3) Simulating the population over 30 years with different turbine mortality and WNS scenarios, and 4) Repeating steps 3 and 4 for 1,000 landscapes. The next section of our manuscript outlines our model using the "Overview, Design concepts, and Details" (ODD) protocol (Grimm et al., 2006, 2010). Although our model is not an Agent Based Model (ABM), the ODD protocol provides a useful framework for documenting our population-level model.

2.1 Purpose

We created this model to describe and understand the effects of wind energy development on the spatial dynamics of the Indiana Bat. We also had to consider the effects of white-nose syndrome upon the species' population dynamics.

2.2 Entities, state variables, and scales

Our model is a full-annual-cycle population model (Hostetler et al., 2015) that tracks groups of bat surviving through four seasons: summer, fall migration, winter, and spring migration. Our state variables are groups of bats that use a specific maternity colony site and hibernaculum. Note the framework was originally developed for avians, hence our choice of Bs for summer sites (or breeding sites in the avian world) and Ns for winter sites (or non-breeding sites in the avian world). Bats are also accounted for by life stages (juveniles/first-year breeders versus adults) and seasonal habitats (maternity sites and hibernacula) during each year, t. This leads to four states variable (here depicted in vector notation): the population of juveniles during the winter season, $N_J(t)$; the population of adults during the winter season, $N_A(t)$; the population of juveniles during the maternity season, $B_J(t)$; and the population of adults during the maternity season,

$B_A(t)$. Each vector's elements depict a specific migratory pathway, e.g., $N_J(t)$ is comprised of $\mathbf{n} \times \mathbf{b}$ elements, $\mathbf{n} \in$ winter sites, $\mathbf{b} \in$ maternity sites. The variables may be summed by either maternity site or hibernaculum to calculate the total population using a specific geographic location. Within our code, we account for this using an index column for maternity sites and an index column for hibernaculum within the data table.

Our choice of state variables caused the time step (i.e. t) to be 1 year. However, we recorded the population of each group during the maternity and winter season as an artifact of our state-variable choice. We choose these state variables partially for their biological information and partially to simplify programming. We ran our simulation for 30 years because the USFWS currently issues Indiana Bat take permits for 30 years.

Our model covers the range of the Indiana Bat, which is approximately the eastern half of the contiguous United States (Figure 1). The boundaries of our range was based upon the United States boundary, the NatureServe Range map, and observations of the species. The maximum migration distance was 500-km, which was based upon field observations reported in the literature (Gardner and Cook, 2002; Winhold and Kurta, 2006). The landscape was covered with approximately 33,000, 6475-ha grid cells and the grid size was based upon management considerations. The U.S. Fish and Wildlife Service considers a 2.5 mile radius around a known maternity colony to be its summer habitat range and all of the hibernaculum within a 2.5 miles radius to be a single management unit. Hence the choice of 5-by-5 square grids (25 miles^2 or 6475 ha). Each group of bats within the model has a summer and winter grid cell as well as a pathway connecting the cells. It is possible for a group to be in the cell for both seasons, but improbable for females (which we modeled). The straight line between summer and winter cells were buffered with different distances (1-km, 2-km, 10-km, 20-km, 100-km, and 200-km) as part of the turbine sensitivity and uncertainty analysis. We dropped the largest two buffer sizes during the model development processes because they were biologically unrealistic and including them caused all populations to go extinct all of the time. Note a 1-km buffer would be a 2-km wide path. An example of two pathways are included in Figure 2. The buffers accounts for bats not migrating in a straight line. If we had precise locations for all summer maternity colonies, other approaches such as Circuitscape (Hanks and Hooten, 2013) could have been used to model migration routes and this would have reduced migration uncertainty.

2.3 Process overview and scheduling

We modeled survival and births during each time step of the model for each life stage. Each model year starts with adults and juveniles surviving the winter and the spring migration and then producing pups (Equation 1) and also surviving and becoming (or staying) adults (Equation 2). Approximately 6 months later, juveniles survive summer and fall migration (Equation 4) and the adults also survive summer and fall migration (Equation 5). The complete set of equations are included in §2.7.2.

2.4 Design concepts

2.4.1 Basic principles

Our population model is a full-annual-cycle population model (Hostetler et al., 2015). We used a series of difference equations (i.e., discrete time) for our model. Our model structure would commonly be referred to as a "population matrix model" by ecologists, however we did not use matrix notation. The model builds

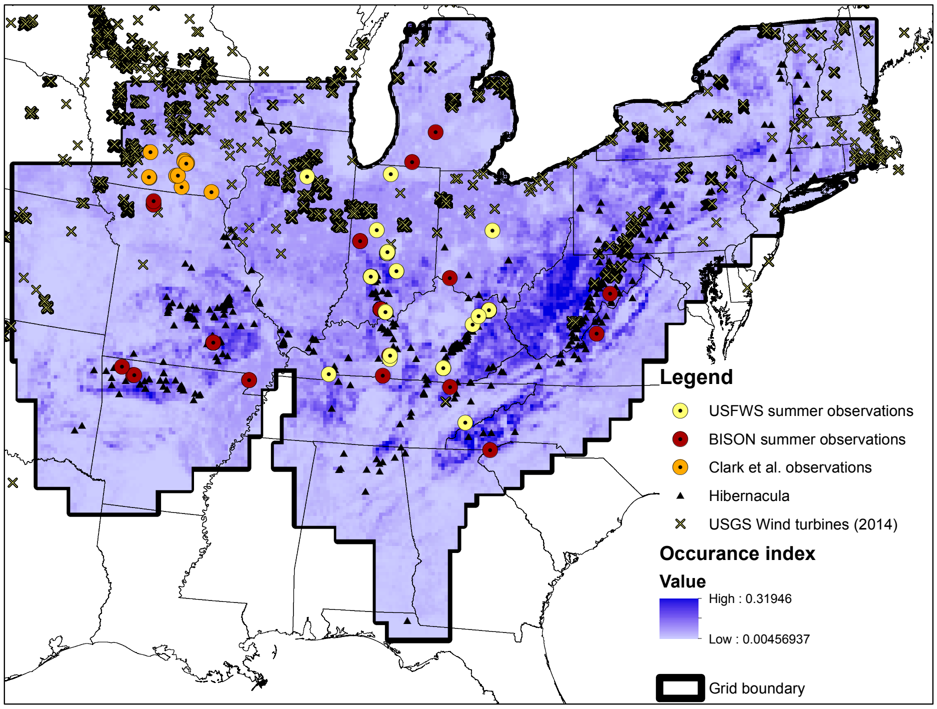

Figure 1: Map of input data, species occurrence map, and Indiana Bat species range. 'USFWS summer observations' are from the US Fish and Wildlife Service. All US Fish and Wildlife Service is from the endangered species program and exact locations are confidential. 'BISON summer observations' are from the Biodiversity Information Serving Our Nation database (BISON; `bison.usgs.ornl.gov`). 'Clark et al. observations' are capture data from Clark et al. (1987). 'Hibernacula' data is the winter hibernacula data from the US Fish and Wildlife Service. 'USGS Wind turbine data (2014)' comes from Diffendorfer et al. (2014). The white-to-blue color gradient depicts low to high suitability from the occurrence model. The grid boundary is the outline of the grid cells used for the occurrence model.

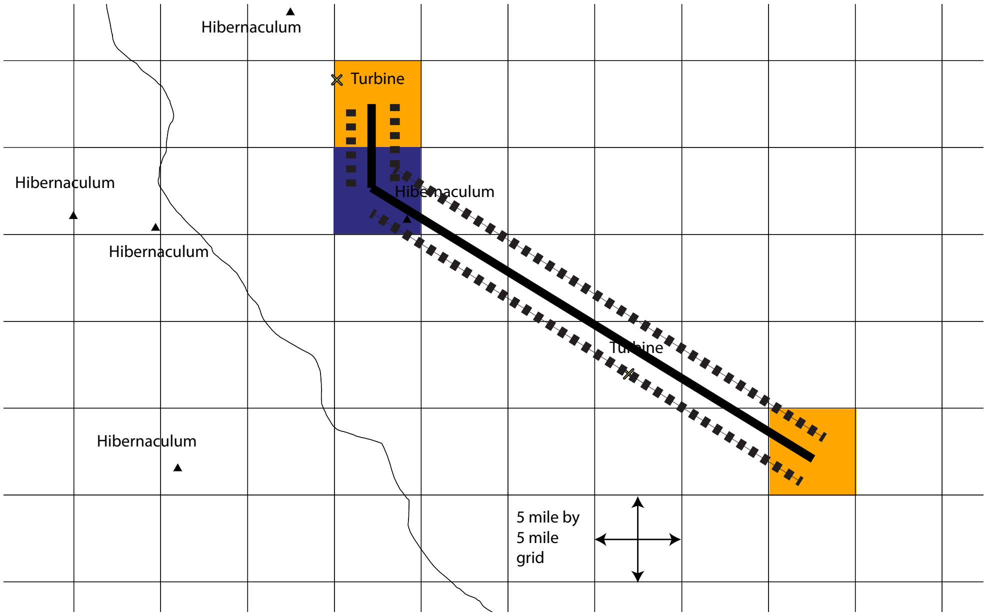

Figure 2: Example landscape illustrating two different pathways. The blue cell contains a hibernacula. The orange cells contains maternity colonies. The migratory pathways are depicted as solid lines and example buffers are dashed lines. The south-to-north pathway would be affected by the wind turbine in the maternity colony cell. The southeast-to-northwest pathway would be affected by the wind turbine inside its buffer area.

upon network theory developed by Taylor and Norris (2010) and Erickson et al. (2014a). We differ from Erickson et al. (2014a) because we do not include density at the hibernacula locations. Otherwise, our model is conceptually identical to Erickson et al. (2014a).

We also include an occurrence model for summer habitat. This model is a logistic regression and follows standard ecological occupancy theory (MacKenzie et al., 2005). Exact model details are included in §2.7.1.

2.4.2 Emergence

Our model does not contain any emergence behavior.

2.4.3 Adaptation

Our model does not include any adaptive behavior.

2.4.4 Objectives

We do not have an adaptive trait with objectives in the current version of the model.

2.4.5 Learning

No bat learning occurs within our model.

2.4.6 Prediction

There is no internal predicting that occurs as part of this model.

2.4.7 Sensing

There is no sensing within the model.

2.4.8 Interaction

Density at the maternity colonies (summer nodes) is included within the model and decreases survival as population size increases.

2.4.9 Stochasticity

Different landscapes are generated, which affects exposure to wind turbines. The demographic parameters are constant because previous work with the model demonstrated that parameter variability has a minimal effect on the dynamics (Erickson et al., 2014b). This observation is also an artifact of the life history of the Indiana Bat. The bat is a long-lived species that only produces 1 pup per year, which limits the parameter possible demographic parameter space.

2.4.10 Collectives

Bats belong to sub-populations that use a migratory pathway between maternity colonies (summer nodes) and hibernacula (winter nodes). Each maternity colony has a density function. Each group may be summed by maternity colony or hibernaculum to calculate the population size using a node.

2.4.11 Observation

We recorded the summer and winter population of bats for each pathway through time, although we only report the total winter population because population sampling is only done for the species at their hibernacula.

2.5 Initialization

The initial state of the model is the placement of bats on the landscape (i.e., which migratory pathways are the bats using?) and is stochastic. The rest of the model is deterministic. Bat groups were placed on the landscape with the `hibDistrib` function in our code. This function divides bats at each hibernaculum into groups and then assigns each group to a maternity colony cell. The probability of Indiana Bats occurring in a cell (See §2.7.1 for details) was multiplied by an exponential decay function that penalized sites farther away from the hibernacula ($e^{-1\times10^{-6}\times\text{distance in meters}}$). This function was based upon the literature (Gardner and

Cook, 2002; Winhold and Kurta, 2006). Maternity colony selection was based upon the quality of site from the habitat model and the distance from the hibernaculum. We assumed a mean group size of 75 bats.

These probabilities were used to place bats on the landscape. The bats at a hibernacula were split into groups assuming a maximum group size of 75 because the USFWS estimates an average colony to range from 50 to 80 adult females with a total of USFWS estimated 2,859 to 4,574 maternity colonies (note this estimate excludes both WNS and the discovery of a new hibernacula in Missouri containing as many 100,000 bats) (Pruitt and TeWinkel, 2007). For example, a hibernaculum with 70 bats would be one group, a hibernaculum with 80 bats would be split into two groups of 40, and a hibernaculum with 1,000 bats would be split into 14 groups, each with 71 bats. We allowed maternity colonies to have mixed populations from multiple hibernacula.

2.6 Input data

We used multiple sources of data for our model. Broadly, our data may be divided into two groups, habitat data used to model the summer habitat of the species and hibernacula locations (§2.6.1) and direct population model inputs (§2.6.2).

2.6.1 Habitat data

Multiple data sources were used for our habitat model for both the predictor and response variables. We had observations of the Indiana bat from three sources for maternity roost sites and the census data for the hibernacula (Figure 1). Our hibernacula data came from the USFWS and is a private data set because hibernacula locations are confidential. We used the hibernacula location data to calculate the total number of hibernacula per grid cell and total population found in a grid cell. We used the maximum observed population over the last 3 years of data to calculate the population size. We choose three years because most hibernacula were sampled ever-other year prior to the arrival of WNS, but some were sampled less frequently. Additionally, most sampling was halted post-WNS in order to limit accidental spread of the disease.

We had banding data from previous banding efforts from the US Fish and Wildlife Service. The banding data comes from a wide variety of sources including academic researchers and a national bat banding project. Additional banding data exists, but has yet to be digitized (Ellison, 2008). The national bat banding project existed from the 1930s-1970s until the program was halted due to a high banding mortality (Ellison, 2008). We also used observation data from the Biodiversity Information Serving Our Nation database (BISON; `bison.usgs.ornl.gov`). This database aggregates primarily museum records from across the United States. For the BISON data, we only included female observations from the summer (June and July). We also excluded an observation from Florida because the population there has been extirpated, the sample was an accidental occurrence, or the sample was misidentified (`http://www.iucnredlist.org/details/14136/0`). We also excluded any data points from Wisconsin because the Indiana Bat has not been observed in the state since 1960 (WI DNR; `http://dnr.wi.gov/topic/EndangeredResources/Animals.asp?mode=detail&SpecCode=AMACC01100`) and the single observation may have actually been a mistaken sample identification (Jackson, 1961). We also used observations from Clark et al. (1987). This data helped us by extending the species range in western Iowa on the northwestern edge of the species' range. All three data sets were aggregated and the presence for each data cell was tabulated.

We used multiple different habitat layers to predict observations of the Indiana Bat. Specific predictor variables we examined included:

- Mean elevation for each grid cell, which came from the LANDFIRE database (http://www.landfire.gov/NationalProductDescriptions7.php)
- Maximum slope for each grid cell, which came from the LANDFIRE database (http://www.landfire.gov/NationalProductDescriptions9.php)
- Percentage crop cover, deciduous forest cover, mixed forest cover, and evergreen cover, which came from the National Land Cover Database 2011 (NLCD 20111; http://www.mrlc.gov/nlcd2011.php)
- Length of rivers and streams in each grid cell, which came from the ESRI SDC layer
- Canopy type/existing vegetation type, which came from the LANDFIRE database (http://www.landfire.gov/NationalProductDescriptions21.php)
- Monthly mean temperate and precipitation for May through August from the PRISM Climate Group (http://prism.oregonstate.edu/)

These were chosen based upon previous research demonstrating importance of habitat and environmental data in predicting where Indiana Bat are found on both local (Farmer et al., 2002; Miller et al., 2002; Yates and Muzika, 2006) and regional scales (Loeb and Winters, 2012; Weber and Sparks, 2013). Although Indiana Bats do not necessarily do well with high-input agriculture because it removes their food (i.e., insects) from the landscape, the species exhibits strong philopatry and may be found in agriculturally intense areas.

2.6.2 Population model inputs

The population model used the results of the habitat model as an input for the initial conditions. We also used the USGS Wind Turbine Data (Diffendorfer et al., 2014). We had the 2014 turbine data in addition to the publicly available data that includes all known turbines up-to and including 2013. Turbines were tallied for each grid cell and each pathway by year using a Python script. Our WNS data came from the USFWS led efforts to monitor and control WNS, www.whitenosesyndrome.org. Specifically, we used the WNS spread Map from 12 March 2015 (https://www.whitenosesyndrome.org/resources/map), which was shared with us the by data's curator, Lindsey Hofferman from the PA Game Commission. This data is semi-privileged and she should be contacted directly for the data.

2.7 Submodels

2.7.1 Habitat occurrence model

We developed a habitat occurrence model for the Indiana Bat's summer range. Our model range was based upon the NatureServe range map (http://www.natureserve.org/conservation-tools/data-maps-tools), but included additional areas of the United States to ensure all of our data points were within grid cells. Specifically, we used a logistic regression. Our effort was necessary because no occurrence models existed at our desired resolution. Our effort is similar to previous work by Loeb and Winters (2012) and Weber and Sparks (2013). Our work differs because Loeb and Winters (2012) and Weber and Sparks (2013) both

modeled the summer maternity roost colonies at the county level, whereas we modeled the species at the resolution of 5-by-5 square mile gird cells (25 sq. miles or 6500 ha). We chose this resolution because the USFWS considers a 2.5 miles radius around a maternity roost to be the habitat used by Indiana Bats from a management perspective.

We initially planned on using a Poisson point process model (Warton and Shepherd, 2010; Renner and Warton, 2013). This approach is one method to account for situations where there are only observations of a species (compared to having true absences data). A Poisson point process is a two or more dimension model that predicts the point occurrence of events (e.g., species observations, eye movements, etc.). The results from logistic regression converge to the results from a Poisson point process model as the number of regularly spaced or randomly chosen points increases (Warton and Shepherd, 2010). This approach requires changing the number of regularly spaced points to identify the optimal resolution. However, we were unable to change points due to the large spatial scale of our data and computational limits of this project. Additionally, the managers using our data were interested in predicting the occurrence of Indiana bat for 5-by-5 mile grids for previously mentioned reasons.

We also examined different landscape variables and their interaction with each other variables. The National Land Cover Database from 2011 (NLCD) crop cover and deciduous forest cover had an interaction with slope (Figure 1). This is reasonable for the eastern United States because most arable land has either been converted to farm land or developed for other human uses. This led us to our final model excluding slope and only including crop cover, deciduous forest cover, and an interaction between the two variables.

We examined several environmental variables in our attempt to predict the occurrence of the Indiana Bat. Forest cover, structure, and roost tree availability are important in predicting locations where Indiana Bats are found on local (Farmer et al., 2002; Miller et al., 2002; Yates and Muzika, 2006) and regional scales (Loeb and Winters, 2012; Weber and Sparks, 2013). Although Indiana Bats do not necessarily do well with high-input agriculture because it removes their food (i.e., insects) from the landscape, the species exhibits strong philopatry.

We used the Widely Applicable Information Criterion (WAIC; also knowns as Watanabe Akaike Information Criterion; Watanabe, 2010) for model selection of variables. We used WAIC because it is a fully Bayesian model selection approach (Gelman et al., 2013). We used Stan (version 2.4, `www.mc-stan.org`), as implemented through RStan, to fit our models and calculated the WAIC values (Hoffman and Gelman, 2014). We compared variables within different types to narrow down variable choices (e.g., we compared different month's climate data to find the "best" temperature and precipitation predictor variable for each month). We used heavily urbanized cells as pseudo-absence cells because Indiana Bats are very rarely found in urban environments. After this selection, we constructed and compared various models to the global model. We then used the results to create a map predicting the observation of Indiana Bats across the landscape. We also compared polynomial regressions ($y \sim \beta_0 + \beta_1 x + \beta_2 x^2 \ldots$) because previous researchers found nonlinear responses (Farmer et al., 2002; Miller et al., 2002; Yates and Muzika, 2006; Loeb and Winters, 2012; Weber and Sparks, 2013) and polynomial regression is a method to capture this salient feature (Harrell, 2001).

We thought climate variables might be important for two reasons (Loeb and Winters, 2012). First, climate may directly affect the distribution of the Indiana Bat, e.g., the Indiana Bat does best under an optimal temperature. Second, temperature may affect the plant communities and therefore the habitat for the Indiana Bat. We examined 30-year mean temperatures and precipitations for the given periods from 1980-

2010. We found that mean May precipitation and mean June temperature best predicted the occurrence of Indiana Bats.

We found the model that included percentage deciduous forest cover (D), percentage crop cover (C), the interaction between the two ($D \times C$), and mean May precipitation (P_{Max}) performed best (Table 1). The model's performance was almost identical to the full model that also included mean June temperature (T_{June}), but the coefficient for this parameter included zero within the 95% posterior interval (-0.33 mean, -0.69 to 0.03). All parameters within the best fitting model were positively associated with increasing probability of observing Indiana Bats in a grid cell (Table 2). The lack of importance of June temperature appears reasonable because we truncated areas to the north or south of the species current range. The interaction term between deciduous forest cover and crop cover likely captures elevation and slope (which were both highly correlated) because much of the land in the Eastern United States is either under cultivation or too steep to farm and therefore forested.

Table 1: Watanabe Akaike Information Criterion (WAIC) values for the final set of models. C is the percentage crop cover in a grid cell, D is the percentage crop cover in a grid cell, $D \times C$ is the interaction between D and C, P_{May} is the mean May precipitation (in mm), and T_{June} is the mean June temperature (in degrees Celsius).

Model	WAIC
Intercept $+ D + C + D \times C + P_{\mathrm{May}}$	206.96
Intercept $+ D + C + D \times C + P_{\mathrm{May}} + T_{\mathrm{June}}$	207.42
Intercept $+ D + C + D \times C$	226.96
Intercept $+ D + C$	236.75
Intercept Only	423.10

Table 2: Parameter estimates for model with the lowest WAIC score (Intercept $+ D + C + D \times C + P_{\mathrm{May}}$). The subscripts of the parameters correspond to the predictor variables (e.g., β_0 is the intercept): 0 is the intercept, C is the percentage crop cover in a grid cell, D is the percentage deciduous forest cover in a grid cell, $D \times C$ is the interaction between D and C and P_{May} is the mean May precipitation (in mm). The 'mean' is the mean from the posterior parameter distribution and it's corresponding standard error ('se_mean'), standard deviation ('sd'), and quantiles (e.g., '2.5%'). The number of effective Monty Carlo samples ('n_eff') and $\hat{r}$ ('Rhat') are also included. Both indicated that the MC samples have converged (Gelman and Rubin, 1992; Gelman et al., 2013).

	mean	se_mean	sd	2.5%	25%	50%	75%	97.5%	n_eff	Rhat
β_0	-18.09	0.11	1.78	-21.67	-19.30	-18.12	-16.78	-14.75	275.13	1.01
β_C	11.39	0.10	1.67	8.34	10.21	11.37	12.39	14.96	281.04	1.01
β_D	14.44	0.11	1.77	11.23	13.24	14.28	15.51	18.16	267.74	1.01
$\beta_{D \times C}$	12.19	0.34	6.24	0.46	7.95	12.07	16.26	24.14	332.02	1.01
$\beta_{P_{\mathrm{May}}}$	0.06	0.00	0.01	0.04	0.06	0.06	0.07	0.09	279.42	1.01

2.7.2 Population model

Our model includes a population model that is an adaptation of two existing models by Thogmartin et al. (2012) and Erickson et al. (2014a). The model is a stage-structured population model following juvenile (first-year breeders) and adult bats at summer sites (B_J and B_A) and hibernacula (N_J and N_A) through time (t). We also kept track of the total population for each season ($B_T(t)$ and $N_T(t)$). The model formulation is identical to the model presented in Erickson et al. (2014a) other than the following changes: 1) we removed

density dependency from the hibernaculum, 2) we removed distance-induced mortality from the migration survival term, and 3) we explicitly keep track of both summer and winter populations. We included the model's equations (Equations 1 to 5), but refer the reader to the original publications for further explanation and discussion of the underlying model (Thogmartin et al., 2012; Erickson et al., 2014a). The equations are a series of difference equations with a discrete time step of 1 year, although the difference between summer and winter seasons would be approximately 6 months. Note that the year changes after the summer ends.

$$\begin{aligned}
B_J(t) &= f_j c_{Js} S_{NJ} N_J(t) + \\
&\quad f_a c_{As} S_{NA} N_A(t) + && (1)\\
B_A(t) &= c_{Js} S_{NJ} N_J(t) + \\
&\quad c_{As} S_{NA} N_A(t) + && (2)\\
B_T(t) &= B_J(t) + B_A(t) && (3)\\
N_J(t+1) &= c_{Jf} S_{BJ} B_J(t) && (4)\\
N_A(t+1) &= c_{Af} S_{BA} B_A(t) && (5)\\
N_T(t) &= N_J(t) + N_A(t) && (6)
\end{aligned}$$

This model follows sub-populations at summer sites ($b \in 1, 2, 3, \ldots$ b) and winter sites ($n \in 1, 2, 3, \ldots$ n), although these subscripts are not included in the above equations to simplify the presentation. Parameter names and associated distributions are listed in Tables 2 and 3. The summer survivals (S_{BJ} and S_{BA}) were both functions of population size at each time step:

$$\begin{aligned}
S_{BJ} &= s_{BJ} e^{B_T(t)/K_b} && (7)\\
S_{BA} &= s_{BA} e^{B_T(t)/K_b}. && (8)
\end{aligned}$$

Life history parameters were based upon those used by Thogmartin et al. (2012) and where chosen to have an annual population growth rate of 1.02 without the density effect (Table 3). The parameters estimates originated with an expert elicitation conducted by the USFWS. The original white-nose model syndrome model used by Thogmartin et al. (2012) had discrete levels of white-nose mortality and included an upper and lower bound of a uniform distribution. We examined the best and worst case scenarios as well as medium-level of mortality scenario. We also used fit a continuous function to the line rather than using a piecewise continuous function. This was the `wnsMor` function within our code.

Uncertainty about mortality from wind turbines was incorporated by running multiple scenarios that included: 2 turbine exposure scenarios, 5 migratory path scenarios, and 3 collision risk scenarios (Table 4). Our exposure scenarios included mortality only along the migratory route and also mortality at the hibernacula and maternity colonies (Figure 4). Uncertainty exists as to the exact migration routes taken by Indiana Bats. To account for this uncertainty, we connected the midpoints of maternity colony cells with the midpoint of the hibernacula cells. This straight line was then buffered by 1-km, 2-km, 10-km, and 20-km and all of the turbines within these buffer areas were enumerated. Our fifth scenario included no turbines in the buffer. We also initially included 100-km and 200-km buffers, but these proved to be too wide to be biologically meaningful. Currently, uncertainty exists as to the probability of a bat being killed by a wind turbine when it flies by the turbine because no bat studies have been done that are similar to work on birds

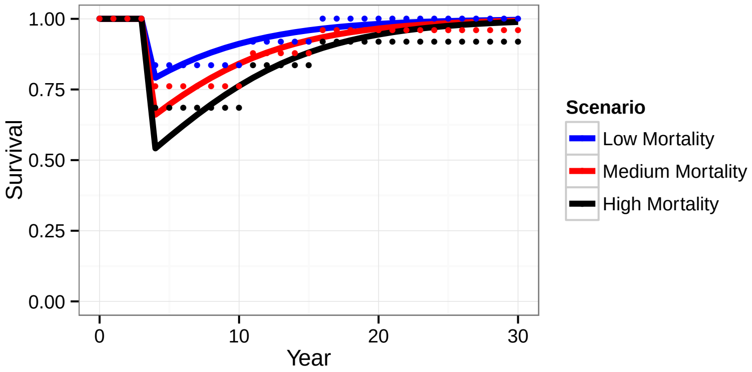

Figure 3: The three white-nose mortality submodels used in our simulations.

Table 3: Parameter values used within population model.

Parameter	Symbol	Value
Adult pup production	J_a	0.45
Baseline adult maternity season survival	s_a^b	0.965
Ricker density term	K_b	2.5×10^{-4}
Adult hibernation survival	s_a^n	0.97
Adult fall migration survival	c_a^f	0.95
Adult spring migration survival	c_a^s	0.95
First-year (juvenile) pup production	J_j	0.25
Baseline first year (juvenile) maternity season survival	s_j^b	0.8685
First-year (juvenile) hibernation season survival	s_j^n	0.873
First-year (juvenile) fall migration survival	c_j^f	0.85
First-year (juvenile) spring migration survival	c_j^s	0.85
First-year (juvenile) pup production	J_j	0.25

by Loss et al. (2013). We accounted for this uncertainty by using three different probabilities of collision: 1) a low risk where 1 out of 1,000 bats flying by a turbine is killed, 2) a medium risk where 1 out of 100 bats flying by a turbine is killed, and 3) a high risk where 1 out of 10 bats flying by a turbine is killed. This risk was used to calculate the decrease in migration survival caused by turbines. As an example, if a path had 10 turbines, the migration survival parameters would become $c \times (1 - 1/100)^{10} = 0.904$ for the medium exposure scenario.

The model was programmed in **R** (R Core Team, 2014). The code was vectorized using the `data.table` package (Dowle et al., 2014). This package was used because of its optimized memory use. We parallelized our code for the stochastic runs using the `doSNOW` package (Analytics and Weston, 2014). We have included our code as a supplement to this document.

3 Data evaluation

This TRACE element provides supporting information on: The quality and sources of numerical and qualitative data used to parameterize the model, both directly and inversely via calibration, and of the observed patterns that were used to design the overall model structure. This critical evaluation will allow

Table 4: Uncertainty in wind turbine exposure scenarios and WNS used within our model.

Uncertainty source	Scenarios
Exposure uncertainty	Only along migratory paths
	Both maternity site/hibernacula and paths
Collision risk uncertainty	Low (1/1,000 bats killed)
	Medium (1/100 bats killed)
	High (1/10 bats killed)
Migration path uncertainty	No turbines in path
	All turbines in a 1-km buffer
	All turbines in a 2-km buffer
	All turbines in a 10-km buffer
	All turbines in a 20-km buffer
WNS uncertainty	No WNS
	Low WNS mortality
	Medium WNS mortality
	High WNS mortality

model users to assess the scope and the uncertainty of the data and knowledge on which the model is based.

Summary:

Our model had multiple sources of data input that are described in §2.6. We discuss the limitations of the quality of our data here.

Portions of this section could be redundant with §2.6. Therefore, we focus data on quality, which was not previously addressed and refer the reader to §2.6 for additional details.

Our habitat model included formal model parameterization and selection that is described in §2.6.1. The observed bat data is the best available data, but is limited in quantity, especially given the large spatial range of the model. The limited number of data points also limited the number of parameters that we used to parameterize the model without over-fitting the data. The predictor variables (e.g., land cover data) are mainly peer-reviewed scientific products of the USGS. Although there are issues related to scaling datasets such as these (e.g., Thogmartin et al., 2004; Saura and Castro, 2007), these usually deal with sub-pixel extrapolation whereas we are dealing with many pixels per grid cell.

Our wind turbine data is the best available data of wind turbine locations (Diffendorfer et al., 2014) (also see §2.6.2). The data is based upon records from the US Federal Aviation Administration and most (99%) of turbines in this dataset have been verified by remote sensing (i.e., they appear on areal or satellite imagery).

Our WNS data is the data best known to the USFWS (see §2.6.2). The data is based upon histological data on bats and molecular confirmation of *Pseudogymnoascus destructans* the fungus causing WNS.

Our bat life history parameter values came from an expert elicitation conducted by the USFWS that was described in by Thogmartin et al. (2012). This elicitation also examined the mortality of WNS to the Indiana Bat. Although there is a great deal of variability in these parameters from the elicitation, this variability is less important than the impacts of WNS (Erickson et al., 2014b).

All portions of our model were limited by the paucity of data on bats, wind turbines, and their interaction. Very few population-level models or studies exist for the any bat species and more specifically, the Indiana Bat. Prior to WNS arriving, no population models existed for any bat species (Hallam and Federico,

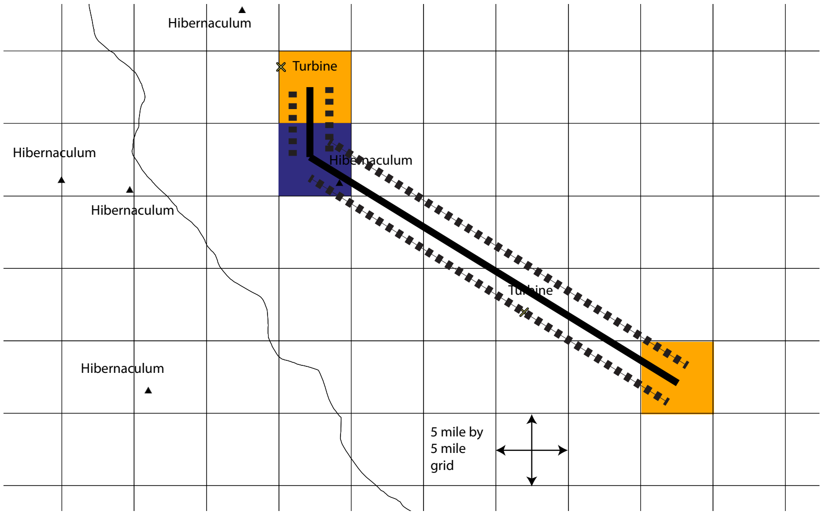

Figure 4: Example of 2 different maternity colonies that share a hibernacula. The northern colony would be affected by 1 turbine during the summer but not migration. The southeastern colony would be affected by 1 turbine during spring and fall migration.

2009). Similar, collision risk data for turbines is sparse. Birds collision risk data is sparse and lacks enough data for species specific modeling (Beston et al., 2015) and bird collisions have been studied much more than bat collisions. For the Indiana Bat specifically, only 5 Indiana Bats have been reported killed to the USFWS though wind energy generation may potentially kill thousands of individuals annually (M. Gannon, Pennsylvania State University, personal communication).

We accounted for this data uncertainty with different scenarios within the model that has been previously described. Unfortunately, not enough data exists for either calibration or integrated population modeling.

4 Conceptual model evaluation

This TRACE element provides supporting information on: The simplifying assumptions underlying a model's design, both with regard to empirical knowledge and general, basic principles. This critical evaluation allows model users to understand that model design was not ad hoc but based on carefully scrutinized considerations.

Summary:

> **Our model captures the spatial dynamics of the Indiana Bat's life cycle (Figure 5). The design concepts and equations are presented in §2. Additionally, the input data and 'big' picture are presented in Figure 1 and the path connections and turbine counts are illustrated in Figure 4.**

Our model captures the salient life history of the Indiana Bat. A literature review of the species life history has been published as part of the species' draft recovery plan (Pruitt and TeWinkel, 2007). The species

produces pups at maternity colonies. These colonies are the density limiting life-stage of the species. However, relatively few observations of summer maternity colonies exist and there is no centralized database of their locations for the Indiana Bat. The bats forage and migrate to hibernacula. Foraging during migration makes bats much different than many species of birds that often fly through without eating along their migration. The exact route bats use for migration is also currently unknown, although in at least some instances, bats appear to follow topography such as rivers and forest edges rather than straight-line migration pathways. We account for this uncertainty by 'buffering' the straight line migration path by different distances. The bats overwinter at the hibernacula. Currently, little is known about the effects of wind energy development on any species of bats, but especially cave bats. We accounted for this by comparing different levels of take within the model.

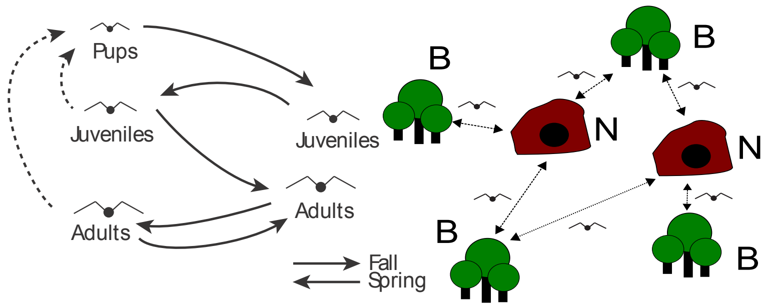

Figure 5: Illustration of how our model contains both stage and spatial structure (reproduced from Erickson et al. (2014a).

5 Implementation verification

This TRACE element provides supporting information on: The simplifying assumptions underlying a model's design, both with regard to empirical knowledge and general, basic principles. This critical evaluation allows model users to understand that model design was not ad hoc but based on carefully scrutinized considerations.

Summary:

> **Our large scale, landscape-level simulations lost tractability due to the scale of the simulation (50,000 paths are hard to visualize!). We conducted small scale simulations that were tractable in order to verify our code. We specifically compared two different landscapes. Both had two maternity sites and two hibernacula. One landscape had 4 paths (i.e., all maternity sites were connected with all hibernacula) and the other had 3 paths.**

We used simplified landscapes to verify our code behaved as expected. Both landscapes had two maternity sites and two hibernacula. Each maternity site had a Ricker density term of 200 (in this simple case, this is also the maternity site's carrying capacity, however, given the non-linear nature of the system, this is not always the case). Both landscapes had 50 bats per path using the non-affected hibernacula (N_4). One landscape had 4 paths (all breeding sites connected to all hibernacula). All paths on this landscape had 50 bats using each path. The other landscape had 4 paths. This landscape only had a path between N_3 and

B_1, but no path between N_4 and B_1. This path had 100 bats using it. The other paths had 50 bats on each of them. All landscapes had a total starting population of 200 bats. We included one turbine on the path between N_3 and B_1 and included no, low (1/1000 bats flying by the turbine), medium (1/100), and high (1/10) levels of mortality from the turbine.

Our results demonstrate the lack of density effects at hibernacula within the model (Figure 6). These results also illustrate the effect of a single turbine on the landscape. A low-level of take had almost no effect, a medium level has a noticeable effect, and a high level may extirpate a subpopulation using a pathway. Our results also demonstrate that maternity sites with multiple paths feeding into them may be able to offset some wind mortality if the populations are currently near their carrying capacities (Figure 6a). Conversely, if a maternity site is only fed by one path, this trend does not emerge (Figure 6b). For a population that has been decimated by stressors such as white-nose syndrome, this outcome would be more likely to occur than on a "healthy" landscape.

6 Model output verification

This TRACE element provides supporting information on: (1) how well model output matches observations and (2) how much calibration and effects of environmental drivers were involved in obtaining good fits of model output and data.

Summary:

> **Our habitat model was fit using model selection criterion and this fit seems reasonable. Our population model was unable to be formally parametrize. However, we were able to check the model's realism and we discuss this qualitative comparison.**

The modeling approach used to fit habitat data has been previously described within this document. Given our knowledge of the species, the most likely habitat for occurrence seems reasonable. The species is most likely to be observed in either the agricultural habitat matrix of the midwest or in the Appalachian mountains of West Virginia. Occasionally, our model places the Indiana Bat in Wisconsin even though the species has never been observed in the state. However, the species is found in the northern lower peninsula of Michigan thus it is somewhat peculiar that it has never been observed in Wisconsin.

The population model outputs seem reasonable. However, the paucity of data on the Indiana Bat limits the model's quantitative fit. We were required to tune the density function of the model. This parameter cannot easily be derived from first principles because we have a non-linear stage-structured model (Allen, 2007). Overall, our model assumptions were reasonable to the USFWS biologists we spoke with about the model given the paucity of data on the species.

7 Model analysis

This TRACE element provides supporting information on: (1) how sensitive model output is to changes in model parameters (sensitivity analysis), and (2) how well the emergence of model output has been understood.

Summary:

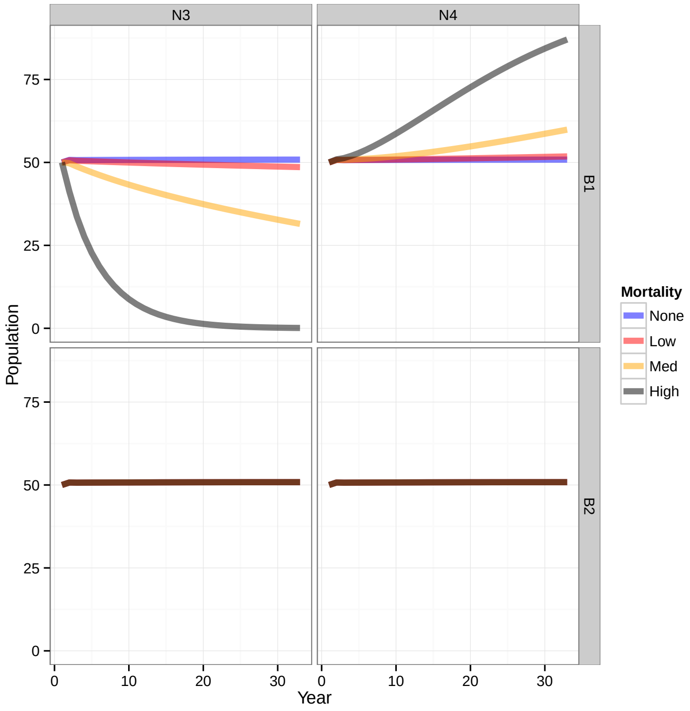

(a) Four-path landscape

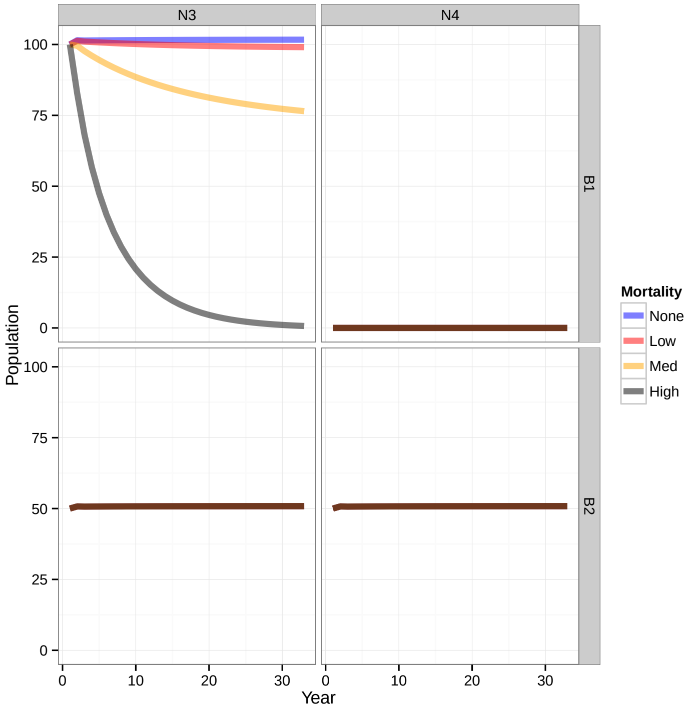

(b) Three-path landscape

Figure 6: Comparison of a four-path (Subfigure 6a) and three-path landscape (Subfigure 6b). Each system had one wind turbine that caused no mortality, low mortality (1/1000 bats flying by killed), medium mortality (1/100), or high mortality (1/10). N are hibernacula sites and B are maternity colonies.

The Indiana Bat is a long-lived species with a low reproductive output. Each female may produce one pup per year as a maximum reproductive output. This constraint makes the population sensitive to even small decreases in survival as illustrated by the wind turbine scenarios. The emerging dynamics of the model largely depend upon where turbines are placed on the landscape. Similar to a game of *Battleship* (Milton Bradley, 1967), some turbine placements may cause more harm to the population than others.

The Indiana Bat is a long-lived species that only produces a maximum of one pup per year. This limits the possible life history parameters and any decrease in parameter values may have an adverse affect on the population size. Previous sensitivity analysis was conducted on the matrix model by Thogmartin et al. (2012). We examined the sensitivity of the model by including wind energy at both non-migratory habitat and along migration paths. We found that current wind farm development was most likely to cause mortality at migratory paths rather than summer or winter habitat. This is likely an artifact given that there is currently little overlap between Indiana Bats and wind turbine development in the eastern United States.

Our findings illustrate how placing wind turbine may be similar to the board game, *Battleship* (Milton Bradley, 1967) where some turbine placements are worse than others. In the game of *Battleship* two players to hit each others ships that are placed on a grid system. Larger ships such as battleships are more likely to be hit because they cover more grid cells than small ships such patrol boats. In a similar manner, migratory pathways also cover a greater portion of the landscape than the summer or winter habitat. The migratory pathways are therefore more likely to have a wind turbine occur within them because they cover a larger portion of the landscape than the other habitats. Additionally, no known hibernacula cells currently have wind turbines located within them. Ultimately though, the paucity of information on the Indiana Bat creates a great deal of uncertainty from our model and the spatial component; more information on the summer habitat use and migratory path choices would help to reduce this uncertainty.

We additionally examined the sensitivity of the mean group size. Our current size falls within the range known by the USFWS as part of the species' habitat conservation plan species' draft recovery plan (Pruitt and TeWinkel, 2007) and the number of summer colonies with the model also corresponds to the number estimated by the USFWS.

Last, the population was sensitive to the WNS induced mortality. We currently do not know how the WNS pandemic will pan out, but it will almost certainly alter the landscape-level abundance of the Indiana Bat.

8 Model output corroboration

This TRACE element provides supporting information on: How model predictions compare to independent data and patterns that were not used, and preferably not even known, while the model was developed, parameterized, and verified. By documenting model output corroboration, model users learn about evidence which, in addition to model output verification, indicates that the model is structurally realistic so that its predictions can be trusted to some degree.

Summary:

Given the landscape and continental-level data that we examined, data that can be

used to corroborate model results is difficult to find. Additional data exists for the Indiana Bat, but has yet to be digitized to a usable format. Our model could be parameterized for a similar *Myotis* spp. However, we chose the Indiana Bat because it is the most highly studied *Myotis* sp. in the United States and because of its status an endangered species.

Bibliography

Allen, L., 2007. An Introduction to Mathematical Biology. Pearson/Prentice Hall, Upper Saddle River, NJ.

Analytics, R., Weston, S., 2014. doSNOW: Foreach parallel adaptor for the snow package. URL: `http://CRAN.R-project.org/package=doSNOW`. R package version 1.0.12.

Beston, J.A., Diffendorfer, J.E., Loss, S., 2015. Insufficient sampling to identify species affected by turbine collisions. The Journal of Wildlife Management 79, 513–517.

Clark, B.K., Bowles, J.B., Clark, B.S., 1987. Summer status of the endangered Indiana bat in Iowa. American Midland Naturalist 118, 32–39.

Diffendorfer, J., Compton, R., Kramer, L., Ancona, Z., Norton, D., 2014. Onshore industrial wind turbine locations for the United States through July, 2013: Data Seriew 817. Technical Report. United States Geological Survey. Denver, CO. URL: `{http://dx.doi.org/10.3133/ds817}`.

Dowle, M., Short, T., Lianoglou, S., with contributions from R Saporta, A.S., Antonyan, E., 2014. data.table: Extension of data.frame. URL: `http://CRAN.R-project.org/package=data.table`. R package version 1.9.4.

Ellison, L., 2008. Summary and analysis of the U.S. goverment Bat Banding Program. Technical Report. US Geological Survey. Washington, DC.

Erickson, R.A., Russell, R.E., Diffendorfer, J.E., Szymanksi, J.A., Thogmartin, W.E., 2014a. A stage-structured, spatially explicit migration model for colonial species with a focus on *Myotis* bats. Letters in Biomathematics 1, 1–16.

Erickson, R.A., Thogmartin, W.E., Szymanski, J.A., 2014b. BatTool: an R package with GUI for assessing the effect of White-nose Syndrome and other take events on *Myotis* spp. of bats. Source Code for Biology and Medicine 9, 9.

Farmer, A.H., Cade, B.S., Stauffer, D.F., 2002. Evaluation of a habitat suitability index model. The Indiana bat: biology and management of an endangered species. A. Kurta and J. Kennedy, eds. Bat Conservation International, Austin, TX , 172–179.

Gardner, J., Cook, E., 2002. Seasonal and geographic distribution and quantification of potential summer habitat. The Indiana bat: biology and management of an endangered species (A. Kurta and J. Kennedy, eds.). Bat Conservation International, Austin, Texas , 9–20.

Gelman, A., Carlin, J.B., Stern, H.S., Dunson, B.D., Vehtari, A., Rubin, D.B., 2013. Bayesian Data Analysis. CRC press, Boca Raton, FL.

Gelman, A., Rubin, D.B., 1992. Inference from iterative simulation using multiple sequences. Statistical Science , 457–472.

Grimm, V., Berger, U., Bastiansen, F., Eliassen, S., Ginot, V., Giske, J., Goss-Custard, J., Grand, T., Heinz, S.K., Huse, G., et al., 2006. A standard protocol for describing individual-based and agent-based models. Ecological Modelling 198, 115–126.

Grimm, V., Berger, U., DeAngelis, D.L., Polhill, J.G., Giske, J., Railsback, S.F., 2010. The ODD protocol: a review and first update. Ecological Modelling 221, 2760–2768.

Hallam, T.G., Federico, P., 2009. Ecological and Behavioral Methods for the Study of Bats. Johns Hopkins University Press, Baltimore. chapter Application of Dynamic Population Models to Bats. pp. 177–194.

Hanks, E.M., Hooten, M.B., 2013. Circuit theory and model-based inference for landscape connectivity. Journal of the American Statistical Association 108, 22–33.

Harrell, F.E., 2001. Regression modeling strategies: with applications to linear models, logistic regression, and survival analysis. Springer.

Hoffman, M.D., Gelman, A., 2014. The no-u-turn sampler: Adaptively setting path lengths in hamiltonian monte carlo. Journal of Machine Learning Research 15, 1351–1381.

Hostetler, J.A., Sillett, T.S., Marra, P.P., 2015. Full-annual-cycle population models for migratory birds. The Auk 132, 433–449.

Jackson, H.H.T., 1961. Mammals of Wisconsin. University of Wisconsin Press.

Loeb, S.C., Winters, E.A., 2012. Indiana bat summer maternity distribution: effects of current and future climates. Ecology and evolution 3, 103–114.

Loss, S.R., Will, T., Marra, P.P., 2013. Estimates of bird collision mortality at wind facilities in the contiguous united states. Biological Conservation 168, 201–209.

MacKenzie, D.I., Nichols, J.D., Royle, J.A., Pollock, K.H., Bailey, L.L., Hines, J.E., 2005. Occupancy Estimation and Modeling: Inferring Patterns and Dynamics of Species Occurrence. Elsevier.

Miller, N., Drobney, R., Clawson, R., Callahan, E., 2002. Summer habitat in northern missouri. The Indiana bat: Biology and management of an endangered species. A. Kurta and J. Kennedy, eds. Bat Conservation International, Austin, TX , 165–171.

Pruitt, L., TeWinkel, L., 2007. Indiana Bat (*Myotis sodalis*) Draft Recovery Plan: First Revision. Technical Report. US Fish and Wildlife Service. Fort Sntelling, MN.

R Core Team, 2014. R: A Language and Environment for Statistical Computing. R Foundation for Statistical Computing. Vienna, Austria. URL: `http://www.R-project.org/`.

Renner, I.W., Warton, D.I., 2013. Equivalence of maxent and poisson point process models for species distribution modeling in ecology. Biometrics 69, 274–281.

Saura, S., Castro, S., 2007. Scaling functions for landscape pattern metrics derived from remotely sensed data: Are their subpixel estimates really accurate? ISPRS Journal of Photogrammetry and Remote Sensing 62, 201–216.

Taylor, C.M., Norris, D.R., 2010. Population dynamics in migratory networks. Theoretical Ecology 3, 65–73.

Thogmartin, W.E., Gallant, A.L., Knutson, M.G., Fox, T.J., Suárez, M.J., 2004. A cautionary tale regarding use of the national land cover dataset 1992. Wildlife Society Bulletin 32, 970–978.

Thogmartin, W.E., King, R.A., Szymanski, J.A., Pruitt, L., 2012. Space-time models for a panzootic in bats, with a focus on the endangered Indiana bat. Journal of Wildlife Diseases 48, 876–887.

Warton, D.I., Shepherd, L.C., 2010. Poisson point process models solve the "pseudo-absence problem" for presence-only data in ecology. The Annals of Applied Statistics 4, 1383–1402.

Watanabe, S., 2010. Asymptotic equivalence of bayes cross validation and widely applicable information criterion in singular learning theory. The Journal of Machine Learning Research 11, 3571–3594.

Weber, T., Sparks, D., 2013. Summer habitat identification of an endangered bat *Myotis sodalis*, across its eastern range of the USA. Journal of Conservation Planning 9, 53–68.

Winhold, L., Kurta, A., 2006. Aspects of migration by the endangered Indiana bat, *Myotis sodalis*. Bat Research News 47, 1–6.

Yates, M., Muzika, R., 2006. Effect of forest structure and fragmentation on site occupancy of bat species in missouri ozark forests. Journal of Wildlife Management 70, 1238–1248.
