# Supplementary material for: Effects of wind energy generation and white-nose syndrome on the viability of the Indiana bat
Source: PeerJ. 2016 Dec 22;4:e2830. doi: 10.7717/peerj.2830 (PMC5183089; doi:10.7717/peerj.2830)
Supplement: Supplemental Information 5 [file peerj-04-2830-s005.pdf]

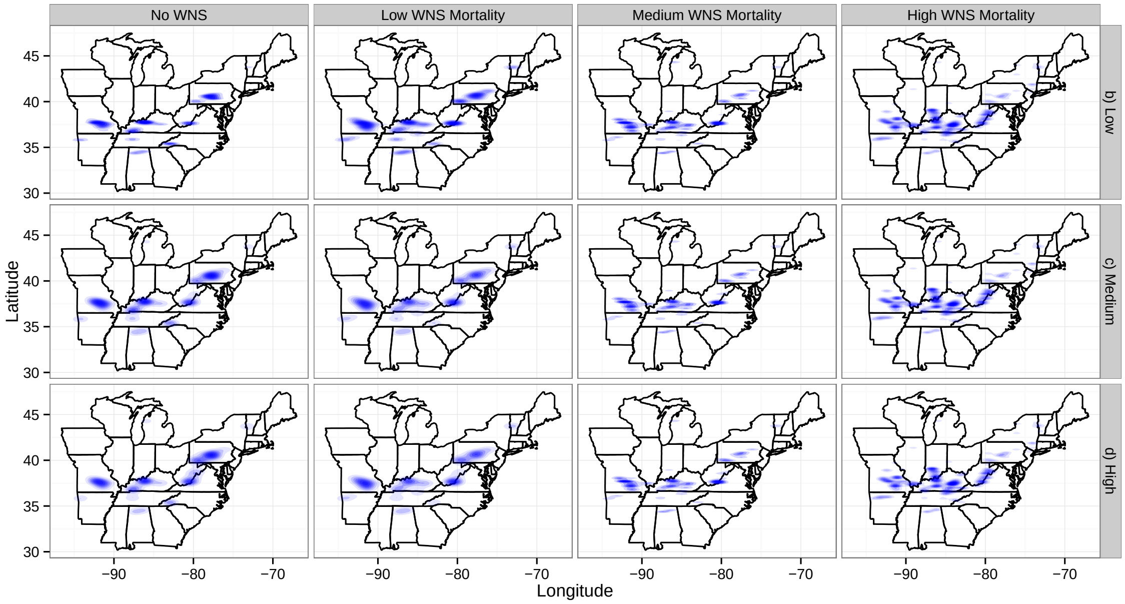

Figure 1: Map of hibernacula lost under different exposure scenarios. The figure is faceted on the x-axis by different WNS mortality scenarios. The figure is faceted on the y-axis by different wind turbine mortality rates. We only show the results from including turbines found within a 2-km buffer the migratory pathway. We also did not plot the scenarios that only included take occurring along migratory pathways. The shading is the relative density of hibernacula lost. The density is subplot specific and only qualitative comparisons should be made across subplots. Also, the densities areas contract as more points are present.

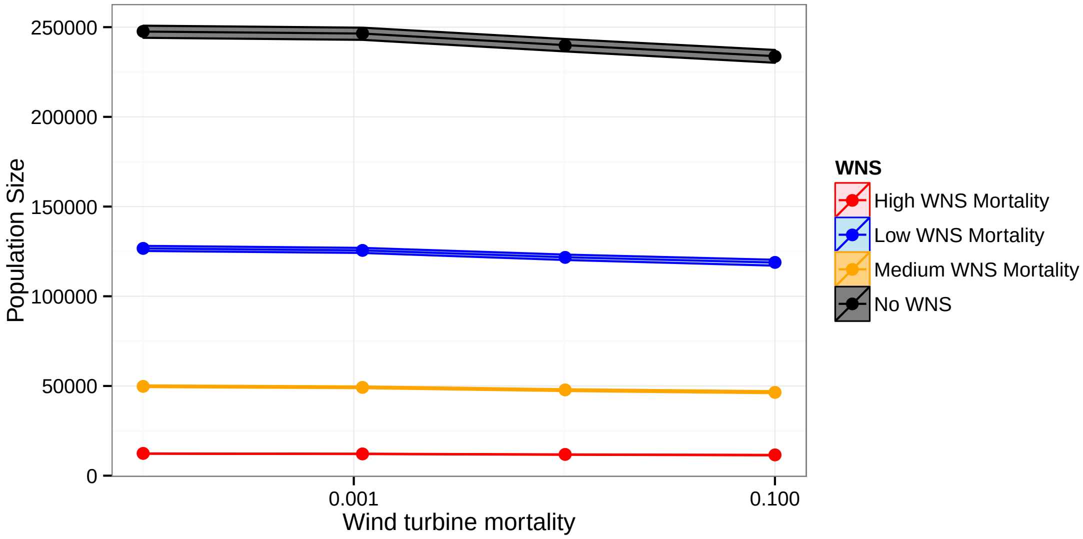

Figure 2: Total population size at the end of the simulation (t = 30 years) plotted against wind energy mortality levels. Note the y-axis is in on the $\log_{10}$ scale and the zero mortality had 1.0×10^{-4} added to it so that it would be transformable. We only show the results from including turbines found within a 2-km buffer the migratory pathway. We also did not plot the scenarios that only included take occurring along migratory pathways.
